# Trends in educational disparities in preventive behaviours, risk perception, perceived effectiveness and trust in the first year of the COVID-19 pandemic in Germany

**DOI:** 10.1186/s12889-022-13341-3

**Published:** 2022-05-06

**Authors:** Timo-Kolja Pförtner, Simone Dohle, Kira Isabel Hower

**Affiliations:** 1 Research Methods Division, Faculty of Human Sciences, University of Cologne, Cologne, Germany; 2 Institute of Medical Sociology, Health Services Research and Rehabilitation Science, Faculty of Human Sciences and Faculty of Medicine, University Hospital Cologne, University of Cologne, Cologne, Germany; 3 Institute of General Practice and Family Medicine, University Hospital Bonn, University of Bonn, Bonn, Germany

**Keywords:** COVID-19, Preventive behaviour, Risk perception, Believed effectiveness, Trust, Educational status

## Abstract

**Background:**

Educational disparities in health and health behaviours have always been relevant in public health research and are particularly challenging in the context of the COVID-19 pandemic. First studies suggest that factors important for the containment of the COVID-19 pandemic, such as prevention behaviour, risk perception, perceived effectiveness of containment measures, and trust in authorities handling the pandemic, vary by educational status. This study builds on recent debate by examining trends in absolute and relative educational disparities in these factors in the first year of the COVID-19 pandemic in Germany.

**Methods:**

Data stem from four waves of the GESIS Panel surveyed between March and October 2020 in Germany (15,902 observations from 4,690 individuals). Trends in absolute and relative disparities were examined for preventive behaviour, risk perception, perceived effectiveness of COVID-19 containment measures, and trust in individuals and institutions handling the COVID-19 pandemic by educational status using sex, age, residence, nationality, children under 16 living in household, family status, household size, the Big Five Inventory, and income class as control factors. Descriptive statistics as well as unadjusted and adjusted linear regression models and random effects models were performed.

**Results:**

We observed an initially rising and then falling trend in preventive behaviour with consistent and significant absolute and relative disparities with a lower preventive behaviour among low educated individuals. Indication of a U-shaped trend with consistent significantly lower values among lower educated individuals was found for risk perception, whereas perceived effectiveness and trust decreased significantly over time but did not significantly vary by educational status.

**Conclusions:**

Results indicate persistent educational disparities in preventive behaviour and risk perception and a general decline in perceived effectiveness and trust in the first year of the COVID-19 pandemic in Germany. To address this overall downward trend and existing disparities, comprehensive and strategic management is needed to communicate the risks of the pandemic and the benefits of COVID-19 containment measures. Both must be adapted to the different needs of educational groups in particular in order to overcome gaps in preventive behaviour and risk perception by educational status.

**Supplementary Information:**

The online version contains supplementary material available at 10.1186/s12889-022-13341-3.

## Introduction

On March 11, 2020, the COVID-19 virus outbreak was officially classified as a pandemic. While more than 118,000 cases from 114 countries and a total of 4,291 deaths had been reported by that date, now more than 350 million cases of infection have been reported, with more than 5 million deaths [1]. The lessons learned from countries such as Italy, France or Spain [2–4], which were particularly hard hit at the beginning of the COVID-19 pandemic in Western countries, led many governments around the world to introduce various public health measures to contain the COVID-19 pandemic. These COVID-19 containment measures ranged from simple recommendations (such as keeping a minimum distance or stay-at-home recommendations) to severe restrictions (such as curfews or closures of educational institutions or of public spaces) [5, 6].

The effectiveness and public acceptance of measures to contain the course, duration, and consequences of a pandemic essentially require large-scale collective action of all citizens within a country. They have a vital role in the successful containment of the pandemic by appropriate preventive behaviours (such as staying at home) and a public support of public containment measures [7–9]. In addition to demographic factors such as age and gender, risk perception, perceived effectiveness of containment measures, and trust in individuals and institutions handling the pandemic play a key role in the effectiveness of public health measures to contain the COVID-19 pandemic [7, 10]. Risk perception is considered a core feature of psychological models of behaviour change, such as the Health Belief Model [11] or the Protection Motivation Theory [12], and is thus an important determinant of cooperation and adoption of preventive behaviours during pandemics [9, 13–15]. Perceived effectiveness and trust are considered crucial factors against detrimental psychological effects of governmental restrictions, and are known to support one's own preventive behaviour and to foster a positive social climate [7, 9, 10, 16, 17]. Recent studies suggest that individuals with low educational status not only have an increased incidence and severity of a COVID-19 infection, but also show less COVID-19 preventive behaviours than others [18]. According to single studies, these educational disparities are also demonstrated in risk perceptions [19], trust [20], and perceived effectiveness of COVID-19 containment measures [21].

**Fig. 1:**
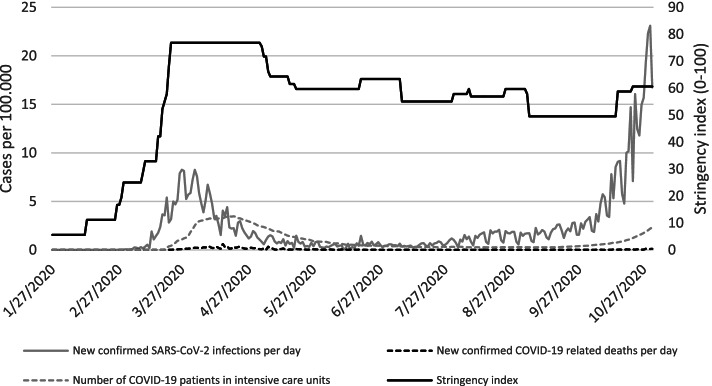
Restriction stringency index, relative confirmed cases of SARS-CoV-2 infections and COVID-19 related deaths, and number of COVID-19 patients in intensive care units in Germany for the data collection period, based on ourworldindata.org. The stringency index is a composite measure based on nine indicators, including school closures, workplace closures, cancellation of public events, restrictions on public gatherings, closures of public transport, stay-at-home requirements, public information campaigns, restrictions on internal movements, and international travel controls, which range from 0 to 100 (100 = most severe) (see: ourworldindata.org)

Describing and explaining educational disparities in health and health behaviours have always been relevant in public health research and is particularly challenging in the context of the COVID-19 pandemic [22–24]. This study builds on this debate by examining trends in absolute and relative disparities in preventive behaviour, risk perception, perceived effectiveness of COVID-19 containment measures, and trust in individuals and institutions by educational status between March and October 2020 in Germany. To better contextualize this study, Fig. 1 provides the reported number of confirmed SARS-CoV-2 infections, COVID-19 related deaths, and COVID-19 patients in intensive care units per day and per 100.000 inhabitants as well as the degree of restrictions in Germany. These factors might correspond to trends in preventive behaviour, risk perception, perceived effectiveness, and trust by educational status, and should be considered when interpreting our findings.

## Methods

### Study design and participants

We used data from the GESIS Panel established in 2013 at the GESIS-Leibniz-Institute for the Social Sciences in Mannheim, Germany. The GESIS Panel is a probability-based mixed-mode access panel that includes a representative sample of the German-speaking population between 18 and 70 years of age with permanent residence in Germany. The initial recruitment was based on random samples of individuals from population registries stratified by regions [25]. Since 2014, the GESIS Panel has been conducted every two months for a duration of 20 min as an online or offline questionnaire (*n* = 4,854 at baseline in 2014, response rate 86.4%) [25]. In 2016 and 2018, the GESIS Panel sample was supplemented by refresher samples taken from samples of the General Population Survey of the Social Sciences (ALLBUS). In 2020, the GESIS Panel comprised approximately 5,000 panellists (a detailed overview of the number of panel participants in the waves included in this study is available in Supplementary Table 1).

The GESIS Panel questionnaires are structured in modules. These modules have different thematic orientations in each of the six annual surveys. The COVID-19 module was introduced initially in the special survey on the Coronavirus SARS-CoV-2 Outbreak in Germany in March 2020 (baseline survey) and were subsequently implemented in three further waves of the survey in May to June 2020 (survey wave 2), July to August 2020 (survey wave 3), and August to October 2020 (survey wave 4). In order to be able to collect data on the pandemic in timely manner, only the subsample of online participants was considered in the special (baseline) survey. In the further survey waves, both online and offline participants were considered. The analytical sample consists of all individuals for which information on the variables considered was available (15,902 observations from 4,690 individuals) (a detailed overview of the total non-response rate and the item unit-nonresponse is available in Supplementary Table 2). The median participation rate was 3.39 observations per person, with 4.6% participating in one (*n* = 217), 8.3% in two (*n* = 389), 30.5% in three (*n* = 1,429), and 56.6% in all survey waves (*n* = 2,655). The characteristics of the analytical sample per survey wave are shown in Table 1.

**Table 1:** Sample characteristics of the analytical sample per survey wave

Survey wave	Baseline	Survey wave 2	Survey wave 3	Survey wave 4
	(March 2020)	(May/June 2020)	(July/August 2020)	(August/October 2020)
	[*n* = 3,016]	[*n* = 4,374]	[*n* = 4,273]	[*n* = 4,239]
Sex, % (n)				
Male	51.5 (1,552)	50.1 (2,190)	50.1 (2,143)	50.3 (2,131)
Female	48.5 (1,464)	49.9 (2,184)	49.9 (2,130)	49.7 (2,108)
Age, mean ± SD	53.9 ± 14.0	56.4 ± 14.2	56.6 ± 14.1	56.4 ± 14.2
Region, % (n)				
West Germany	76.9 (2,318)	74.5 (3,257)	74.1 (3,166)	74.4 (3,154)
East Germany	23.1 (698)	25.5 (1,117)	25.9 (1,107)	25.6 (1,085)
Nationality				
German	97.5 (2,941)	97.6 (4,271)	97.7 (4,174)	97.6 (4,137)
Others	2.5 (75)	2.4 (103)	2.3 (99)	2.4 (102)
Children under 16 living in household, % (n)				
No	74.4 (2,243)	78.0 (3,413)	78.2 (3,340)	77.8 (3,297)
Yes	25.6 (773)	22.0 (961)	21.8 (933)	22.2 (942)
Family status, % (n)				
Single	22.5 (677)	20.2 (883)	19.7 (843)	19.9 (843)
Married	64.2 (1,935)	63.6 (2,782)	64.1 (2,739)	63.9 (2,709)
Divorced/widowed	13.4 (404)	16.2 (709)	16.2 (691)	16.2 (687)
Household size, % (n)				
1-person	11.0 (333)	13.1 (573)	13.0 (555)	12.8 (542)
2-persons	50.1 (1,512)	52.3 (2,289)	52.5 (2,242)	52.4 (2,223)
3-persons	17.7 (533)	15.9 (696)	15.9 (679)	16.0 (680)
4 or more persons	21.2 (638)	18.7 (816)	18.6 (797)	18.7 (794)
Big Five Inventory				
Extraversion mean ± SD	2.7 ± 0.9	2.7 ± 0.9	2.7 ± 0.9	2.7 ± 0.9
Agreeableness mean ± SD	2.7 ± 0.7	2.7 ± 0.7	2.7 ± 0.7	2.7 ± 0.7
Conscientiousness mean ± SD	3.4 ± 0.7	3.4 ± 0.7	3.4 ± 0.7	3.4 ± 0.7
Neuroticism mean ± SD	2.3 ± 0.8	2.3 ± 0.8	2.3 ± 0.8	2.3 ± 0.8
Open-Mindedness mean ± SD	2.9 ± 0.9	2.9 ± 0.9	2.9 ± 0.9	2.9 ± 0.9
Income, % (n)				
Low	23.7 (714)	29.9 (1,310)	30.2 (1,291)	29.9 (1,267)
Intermediate	38.4 (1,160)	38.1 (1,665)	37.8 (1,617)	37.6 (1,596)
High	37.9 (1,142)	32.0 (1,399)	31.9 (1,365)	32.5 (1,376)
Educational status, % (n)				
Low	11.1 (333)	17.8 (777)	17.9 (765)	17.4 (738)
Intermediate	31.2 (942)	33.4 (1,461)	33.7 (1,441)	33.5 (1,419)
High	57.7 (1,741)	48.8 (2,136)	48.4 (2,067)	49.1 (2,082)

### Measurements

#### Dependent variables

Preventive behaviour was measured by asking individuals which measures they have taken in the last seven days. To answer this question, the respondents were given a multiple choice of nine different measures (response category: no vs. yes.) (the exact wording of all items is available in Supplementary Table 3). All items were combined into an additive scale that ranges from 0 to 100, where higher values indicate higher preventive behaviour. Internal consistency measured by the Kuder-Ricardson 20 formula proved to be adequate in each survey wave (KR 20 > 0.5).

Risk perception was measured by a sum score of five items. These capture the respondents' assessment of the likelihood that they or someone in their immediate environment would become infected with SARS-CoV-2, would need hospitalization due to a SARS-CoV-2 infection, would need to be in quarantine, or would infect other persons in the next 24 months (ranging from 1: “not at all likely” to 7: “absolutely likely”) (the exact wording of all items is available in Supplementary Table 4). All items were combined into an additive scale that ranges from 0 to 100, where higher values indicate higher perceptions of risks. The internal consistency measured by Cronbach’s alpha proved to be high in each survey wave (α > 0.8).

Perceived effectiveness of containment measures against SARS-CoV-2 infections was measured by a sum score of seven items. Respondents were asked to rate the effectiveness of measures such as the closure of various facilities and businesses, the ban on visiting hospitals and long-term care facilities, and exit restrictions in containing the COVID-19 pandemic (ranging from: 1 “not effective at all” to 5: “very effective”) (the exact wording of all items is available in Supplementary Table 5). All items were combined into an additive scale that ranges from 0 to 100, where higher values indicate higher perceived effectiveness of COVID-19 containment measures. The internal consistency measured by Cronbach’s alpha proved to be high in each survey wave (α > 0.8).

Trust in people and institutions to handle the COVID-19 pandemic was measured by a sum score of nine items. Respondents were asked to evaluate how much they trust general practitioners, local health authority, municipal and city administration, the Robert Koch Institute (German federal government agency and research institute for disease control and prevention), the federal chancellor, the federal government, the federal ministry of health, the World Health Organization, and scientists handling coronavirus (ranging from 1 “do not trust at all” to 5 “trust completely”) (the exact wording of all items is available in Supplementary Table 6). All items were combined into an additive scale that ranges from 0 to 100, where higher values indicate higher levels of trust in people and institutions to handle the COVID-19 pandemic. The internal consistency measured by Cronbach’s alpha proved to be high in each survey wave (α > 0.8).

#### Independent variable and covariates

Educational status was measured using the ISCED-97 scale [26]. We distinguish between low (ISCED 0–2), intermediate (ISCED 3), and high educational status (ISCED 4–6) in accordance with previous studies [27]. The information on educational status was taken from the last available survey wave, in which the educational status of the panellists was collected. The analyses controlled individual differences in factors that were known to relate to the dependent variables and educational status: sex, age, residence (East or West Germany), nationality (German, others), children under 16 living in household (no, yes), family status (unmarried, married or in partnership or widowed/divorced), household size (one, two, three or four or more persons), the Big Five Inventory (extraversion, agreeableness, conscientiousness, Neuroticism, and Open-Mindedness), and income class [15, 17, 18, 28].

### Statistical analyses

The statistical analyses begin with a descriptive presentation of raw mean values of preventive behaviour, risk perception, perceived effectiveness, and trust for each survey wave stratified by educational status. raw mean values with 95% CIs are presented in Table 2. We tested for educational disparities in preventive behaviour, risk perception, perceived effectiveness, and trust for each survey wave by bivariate linear regression model. To identify the significance of linear or curvilinear trends of preventive behaviour, risk perceptions, perceived effectiveness, and trust, we fitted a random effects panel model with a linear and quadratic trend variable for the total sample and for each educational status group separately. The benefit of random effect modelling is the ability to control for serial correlation of the unobserved characteristics of each individual via time [29]. Therefore, the estimated standard errors will be corrected for the panel structure of the data.

**Table 2:** Raw mean level of preventive behaviour, risk perception, perceived effectiveness, and trust for the total sample and different educational groups by survey wave

Survey wave	**Baseline survey**			**Survey wave 2**			**Survey wave 3**			**Survey wave 4**			P-Trend^b^	P-Trend^b^
	(March 2020)			(May/June 2020)			(July/August 2020)			(August/October 2020)			(linear)	(quadratic)
	Mean	95%-CI	P-value^a^	Mean	95%-CI	P-value^a^	Mean	95%-CI	P-value^a^	Mean	95%-CI	P-value^a^		
**Preventive behaviour**														
Educational status														
Low	47.9	46.0–49.8	Ref	59.8	58.5–61.1	Ref	54.9	53.6–56.2	Ref	53.6	52.0–54.7	Ref	< 0.001	< 0.001
Intermediate	50.1	49.1–51.1	0.031	62.1	61.3–63.0	0.002	55.4	54.5–56.4	0.516	53.6	52.7–54.6	0.749	< 0.001	< 0.001
High	52.4	51.6–53.1	< 0.001	63.6	62.9–64.3	< 0.001	57.0	56.2–57.7	0.007	56.4	55.6–57.2	< 0.001	< 0.001	< 0.001
* Total*	*51.2*	*50.6–51.8*		*62.4*	*61.9–63.0*		*56.1*	*55.5–56.6*		*55.0*	*54.4–55.5*		< *0.001*	< *0.001*
**Risk perception**														
Educational status														
Low	46.4	44.5–48.4	Ref	35.5	34.3–36.8	Ref	35.0	33.8–36.2	Ref	36.2	35.0–37.4	Ref	< 0.001	< 0.001
Intermediate	49.3	48.1–50.4	0.009	38.5	37.5–39.5	< 0.001	37.1	36.2–38.0	0.003	37.6	36.7–38.6	0.056	< 0.001	< 0.001
High	52.0	51.2–52.7	< 0.001	40.6	39.9–41.3	< 0.001	38.8	38.1–39.5	< 0.001	40.0	39.3–40.7	< 0.001	< 0.001	< 0.001
* Total*	*50.5*	*49.9–51.1*		*39.0*	*38.5–39.5*		*37.5*	*37.0–38.0*		*38.6*	*38.0–39.1*		< *0.001*	< *0.001*
**Perceived effectiveness**														
Educational status														
Low	78.4	76.5–80.3	Ref	64.0	62.6–65.4	Ref	59.8	58.4–61.2	Ref	49.9	48.6–51.3	Ref	< 0.001	0.009
Intermediate	80.2	79.1–81.3	0.103	62.8	51.7–63.8	0.146	57.3	56.3–58.4	0.006	46.9	45.9–47.9	0.001	< 0.001	< 0.001
High	78.7	77.9–79.5	0.782	63.7	62.8–64.5	0.673	59.4	58.5–60.2	0.639	50.2	49.3–51.0	0.762	< 0.001	< 0.001
* Total*	*79.1*	*78.5–79.7*		*63.4*	*62.8–64.0*		*58.8*	*58.2–59.4*		*49.0*	*48.4–49.6*		< *0.001*	< *0.001*
**Trust**														
Educational status														
Low	72.8	70.8–74.8	Ref	68.1	66.7–69.5	Ref	68.8	67.3–70.2	Ref	65.0	63.5–66.4	Ref	< 0.001	0.157
Intermediate	72.3	71.2–73.5	0.649	67.2	66.2–68.2	0.265	67.3	66.2–68.3	0.087	64.0	63.0–65.1	0.288	< 0.001	0.001
High	72.6	71.8–73.3	0.784	68.5	67.7–69.2	0.661	68.7	67.9–69.5	0.970	66.5	65.7–67.3	0.066	< 0.001	< 0.001
* Total*	*72.5*	*71.9–73.1*		*68.0*	*67.4–68.5*		*68.2*	*67.7–68.8*		*65.4*	*64.8–66.0*		< *0.001*	< *0.001*

^a^ based on a t-test for mean level differences by educational status

^b^ based on random-effects model for testing linear and quadratic trend in outcome for each educational group separately

*Ref* reference category

**Fig. 2:**
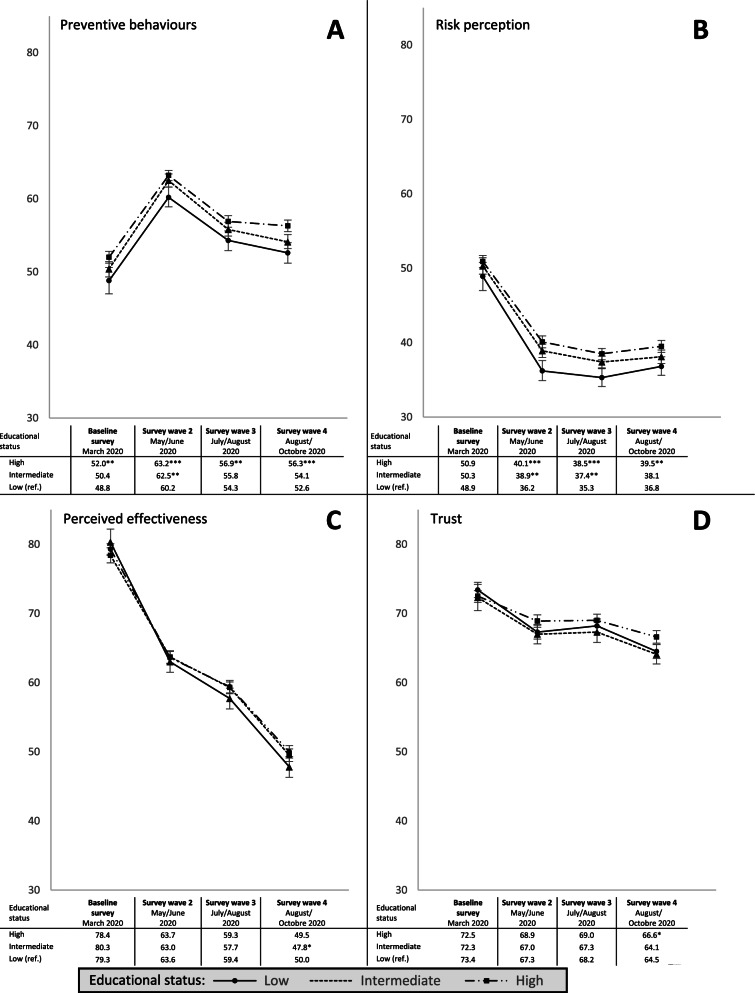
Adjusted marginal means of preventive behaviour (**A**), risk perception (**B**), perceived effectiveness (**C**), and trust (**D**) by survey wave and educational status with 95%-Confidence Interval. Adjusted marginal means were based on linear regression models per survey wave including age, sex, region, nationality, children under 16 years in household, family status, household size, the big five inventory, and income class as control variables. Ref.: reference category. *p < 0.05; **p < 0.01; ***p < 0.001

In a second step, marginal mean values of preventive behaviour, risk perception, perceived effectiveness, and trust were estimated by educational status and for each survey wave separately adjusted for sex, age, residence, nationality, children under 16 living in household, family status, household size, the Big Five Inventory, and income class (Fig. 2). The estimation of the adjusted marginal mean values was based on linear regression models. We also estimated random effects model for each educational status group including all covariates and a linear and quadratic trend variable to test for linear and quadratic trends of preventive behaviour, risk perception, perceived effectiveness, and trust.

In a third step, we quantified the absolute and relative disparities in preventive behaviour, risk perception, perceived effectiveness, and trust by educational status and their trend over time using the Slope Index of Inequality (SII) and Relative Index of Inequality (RII) (Table 3 & 4). The SII represents an absolute difference in outcomes between the lowest and highest educational groups. The RII represents relative disparities in terms of the percentage difference from the population mean in the outcomes between the highest and lowest educational groups. To calculate the SII and RII, educational status was transformed into ridit scores, ranging from 0 (lowest educational status) to 1 (highest educational status). Two-sided 95% CIs for the RII and SII were estimated based on random effects panel modelling. Stepwise regression was used to examine factors affecting relative and absolute disparities in preventive behaviour, risk perception, perceived effectiveness, and trust by educational status. First, educational status, a linear and quadratic trend variable, and an interaction term between educational status and the trend variables was fitted to the data in regression model 1 (M1). Model 2 (M2) was based on M1, and also included the Big Five Inventory to test whether personal traits influence the association between educational status and the outcome measures. Model 3 (M3) was based on M2 and also included sex, age, residence, nationality, children under 16 living in household, family status, and household size, which are assumed to interact with educational status and the outcome measures. Finally, model 4 (M4) was based on M3 and also included income class, which strongly relates to educational status and might also influence educational disparities in preventive behaviour, risk perception, perceived effectiveness, and trust. The degree of model fit was assessed with McFadden’s pseudo R^2^. McFadden’s R^2^ ranges from 0 to 1, with higher values indicating a better model fit. Values between 0.2 and 0.4 are considered to be indicative of excellent model fit. Analyses were performed with Stata 16.1 (StataCorp, Texas, USA).

**Table 3:** Random effects model for absolute educational disparities (SII) in preventive behaviours, risk perception, perceived effectiveness, and trust (Number of observations: 15,902; number of individuals: 4,690)

	M1	M2	M3	M4
	beta (95%-CI)	beta (95%-CI)	beta (95%-CI)	beta (95%-CI)
**Preventive behaviours**				
SII	6.565**	6.779**	7.231**	6.055**
	(2.131,10.998)	(2.337,11.222)	(2.779,11.683)	(1.566,10.544)
Trend (linear)	10.59***	10.52***	10.58***	10.61***
	(8.960,12.230)	(8.886,12.155)	(8.942,12.208)	(8.980,12.247)
Trend (quadratic)	-3.343***	-3.324***	-3.335***	-3.344***
	(-3.827,-2.860)	(-3.808,-2.840)	(-3.818,-2.852)	(-3.827,-2.861)
SII*Trend (linear)	-1.037	-0.967	-1.008	-1.050
	(-3.631,1.556)	(-3.561,1.626)	(-3.599,1.583)	(-3.641,1.541)
SII*Trend (quadratic)	0.316	0.296	0.303	0.313
	(-0.463,1.095)	(-0.483,1.075)	(-0.475,1.082)	(-0.466,1.091)
${R}^{2}$	0.032	0.042	0.077	0.079
${\widehat{\sigma }}_{u}$	13.069	12.928	12.519	12.494
${\widehat{\sigma }}_{e}$	12.210	12.210	12.210	12.210
$\widehat{\rho }$	0.53	0.53	0.51	0.51
**Risk perception**				
SII	11.23***	10.44***	9.443***	8.749***
	(7.046,15.420)	(6.242,14.636)	(5.222,13.663)	(4.491,13.007)
Trend (linear)	-10.81***	-10.80***	-10.72***	-10.68***
	(-12.353,-9.267)	(-12.344,-9.259)	(-12.265,-9.180)	(-12.223,-9.138)
Trend (quadratic)	2.617***	2.615***	2.597***	2.587***
	(2.160,3.073)	(2.158,3.071)	(2.140,3.053)	(2.131,3.044)
SII*Trend (linear)	-2.440	-2.432	-2.488*	-2.543*
	(-4.888,0.007)	(-4.879,0.015)	(-4.935,-0.042)	(-4.990,-0.096)
SII*Trend (quadratic)	0.519	0.517	0.528	0.541
	(-0.217,1.254)	(-0.218,1.252)	(-0.207,1.263)	(-0.194,1.276)
${R}^{2}$	0.080	0.088	0.098	0.099
${\widehat{\sigma }}_{u}$	12.392	12.306	12.195	12.176
${\widehat{\sigma }}_{e}$	11.500	11.500	11.500	11.500
$\widehat{\rho }$	0.54	0.53	0.53	0.53
**Perceived effectiveness**				
SII	-0.145	-0.269	0.217	-0.688
	(-5.036,4.747)	(-5.174,4.636)	(-4.716,5.150)	(-5.662,4.285)
Trend (linear)	-13.67***	-13.70***	-13.69***	-13.67***
	(-15.487,-11.858)	(-15.514,-11.885)	(-15.502,-11.874)	(-15.482,-11.853)
Trend (quadratic)	1.276***	1.283***	1.281***	1.276***
	(0.739,1.812)	(0.746,1.819)	(0.744,1.817)	(0.739,1.813)
SII*Trend (linear)	0.0764	0.105	0.101	0.085
	(-2.802,2.955)	(-2.774,2.983)	(-2.777,2.980)	(-2.794,2.963)
SII*Trend (quadratic)	0.291	0.283	0.283	0.286
	(-0.574,1.156)	(-0.582,1.148)	(-0.582,1.148)	(-0.578,1.151)
${R}^{2}$	0.080	0.088	0.098	0.099
${\widehat{\sigma }}_{u}$	12.392	12.306	12.195	12.176
${\widehat{\sigma }}_{e}$	11.500	11.500	11.500	11.500
$\widehat{\rho }$	0.54	0.53	0.53	0.53
**Trust**				
SII	-1.718	-1.806	0.962	-0.851
	(-5.543,2.107)	(-5.636,2.024)	(-2.904,4.827)	(-4.777,3.075)
Trend (linear)	-4.028***	-4.059***	-4.118***	-4.099***
	(-5.343,-2.713)	(-5.374,-2.744)	(-5.432,-2.803)	(-5.413,-2.784)
Trend (quadratic)	0.423*	0.431*	0.446*	0.441*
	(0.035,0.811)	(0.043,0.819)	(0.057,0.834)	(0.053,0.830)
SII*Trend (linear)	1.320	1.353	1.394	1.377
	(-0.764,3.404)	(-0.731,3.436)	(-0.690,3.477)	(-0.706,3.461)
SII*Trend (quadratic)	-0.005	-0.015	-0.025	-0.021
	(-0.630,0.620)	(-0.639,0.610)	(-0.649,0.600)	(-0.646,0.604)
${R}^{2}$	0.014	0.044	0.065	0.069
${\widehat{\sigma }}_{u}$	16.339	16.004	15.757	15.711
${\widehat{\sigma }}_{e}$	9.729	9.729	9.729	9.729
$\widehat{\rho }$	0.74	0.73	0.72	0.72

M1: SII with linear and quadratic trend variable and an interaction between SII and the linear and quadratic trend variable. M2: M1 + the big five inventory. M3: M2 + sex, age, region, nationality, children under 16 in household, family status, and household size. M4: M3 + income class. ${\widehat{\sigma }}_{u}$: between-unit standard deviation. ${\widehat{\sigma }}_{e}$: within-unit standard deviation. $\widehat{\rho }$: proportion of variance explained by between-unit differences

^*^p < 0.05

^**^p < 0.01

^***^p < 0.001

**Table 4:** Random effects model for relative educational disparities (RII) in preventive behaviours, risk perception, perceived effectiveness, and trust (Number of observations: 15,902; number of individuals: 4,690)

	M1	M2	M3	M4
	beta (95%-CI)	beta (95%-CI)	beta (95%-CI)	beta (95%-CI)
**Preventive behaviours**				
RII	20.09***	20.46***	21.20***	19.03***
	(12.354,27.826)	(12.708,28.213)	(13.425,28.965)	(11.193,26.866)
Trend (linear)	5.350***	5.222***	5.316***	5.384***
	(2.504,8.197)	(2.375,8.068)	(2.472,8.160)	(2.539,8.228)
Trend (quadratic)	-1.426***	-1.393**	-1.412**	-1.428***
	(-2.268,-0.584)	(-2.235,-0.550)	(-2.254,-0.571)	(-2.269,-0.586)
RII*Trend (linear)	-6.706**	-6.584**	-6.655**	-6.728**
	(-11.222,-2.190)	(-11.100,-2.069)	(-11.166,-2.144)	(-11.240,-2.217)
RII*Trend (quadratic)	1.842**	1.808**	1.820**	1.837**
	(0.486,3.198)	(0.451,3.164)	(0.465,3.175)	(0.481,3.192)
${R}^{2}$	0.005	0.016	0.052	0.054
${\widehat{\sigma }}_{u}$	23.192	22.944	22.223	22.176
${\widehat{\sigma }}_{e}$	21.241	21.241	21.241	21.241
$\widehat{\rho }$	0.54	0.54	0.52	0.52
**Risk perception**				
RII	16.53**	14.51**	12.41*	10.79*
	(6.146,26.908)	(4.108,24.920)	(1.942,22.877)	(0.223,21.350)
Trend (linear)	1.706	1.726	1.904	2.002
	(-2.110,5.523)	(-2.089,5.542)	(-1.912,5.719)	(-1.814,5.819)
Trend (quadratic)	-0.322	-0.327	-0.367	-0.39
	(-1.451,0.807)	(-1.456,0.802)	(-1.496,0.762)	(-1.519,0.739)
RII*Trend (linear)	0.366	0.388	0.259	0.127
	(-5.688,6.420)	(-5.665,6.441)	(-5.793,6.311)	(-5.926,6.179)
RII*Trend (quadratic)	-0.251	-0.256	-0.23	-0.199
	(-2.069,1.567)	(-2.074,1.562)	(-2.048,1.588)	(-2.016,1.619)
${R}^{2}$	0.009	0.018	0.028	0.0290
${\widehat{\sigma }}_{u}$	31.292	31.075	30.811	30.765
${\widehat{\sigma }}_{e}$	28.441	28.441	28.441	28.441
$\widehat{\rho }$	0.55	0.54	0.54	0.54
**Perceived effectiveness**				
RII	-2.953	-3.226	-2.339	-3.793
	(-11.274,5.368)	(-11.571,5.120)	(-10.733,6.054)	(-12.257,4.671)
Trend (linear)	-0.778	-0.812	-0.797	-0.764
	(-3.861,2.305)	(-3.895,2.271)	(-3.880,2.287)	(-3.848,2.319)
Trend (quadratic)	-0.197	-0.188	-0.191	-0.198
	(-1.109,0.716)	(-1.100,0.725)	(-1.103,0.722)	(-1.111,0.714)
RII*Trend (linear)	1.414	1.45	1.448	1.421
	(-3.477,6.306)	(-3.441,6.342)	(-3.444,6.339)	(-3.471,6.313)
RII*Trend (quadratic)	0.303	0.293	0.292	0.298
	(-1.167,1.772)	(-1.176,1.763)	(-1.177,1.762)	(-1.172,1.767)
${R}^{2}$	0.001	0.002	0.010	0.011
${\widehat{\sigma }}_{u}$	23.487	23.466	23.326	23.311
${\widehat{\sigma }}_{e}$	23.018	23.018	23.018	23.018
$\widehat{\rho }$	0.51	0.51	0.51	0.51
**Trust**				
RII	-4.287	-4.401	-0.288	-2.934
	(-9.890,1.316)	(-10.011,1.209)	(-5.950,5.374)	(-8.686,2.818)
Trend (linear)	-1.592	-1.637	-1.722	-1.695
	(-3.515,0.331)	(-3.559,0.285)	(-3.645,0.200)	(-3.617,0.227)
Trend (quadratic)	0.104	0.116	0.137	0.131
	(-0.464,0.672)	(-0.452,0.683)	(-0.431,0.705)	(-0.437,0.698)
RII*Trend (linear)	2.853	2.900	2.959	2.936
	(-0.195,5.900)	(-0.147,5.946)	(-0.087,6.006)	(-0.110,5.982)
RII*Trend (quadratic)	-0.201	-0.214	-0.229	-0.224
	(-1.115,0.713)	(-1.128,0.699)	(-1.143,0.685)	(-1.138,0.690)
${R}^{2}$	0.001	0.031	0.052	0.057
${\widehat{\sigma }}_{u}$	24.074	23.584	23.217	23.150
${\widehat{\sigma }}_{e}$	14.225	14.225	14.225	14.225
$\widehat{\rho }$	0.74	0.73	0.73	0.73

M1: RII with linear and quadratic trend variable and an interaction between RII and the linear and quadratic trend variable. M2: M1 + the big five inventory. M3: M2 + sex, age, region, nationality, children under 16 in household, family status, and household size. M4: M3 + income class. ${\widehat{\sigma }}_{u}$: between-unit standard deviation. ${\widehat{\sigma }}_{e}$: within-unit standard deviation. $\widehat{\rho }$: proportion of variance explained by between-unit differences

^*^p < 0.05

^**^p < 0.01

^***^p < 0.001

## Results

Raw mean levels and trends in preventive behaviour, risk perception, perceived effectiveness, and trust by educational status were shown in Table 2. For preventive behaviour, we observed a significant inverted U-shape trend for all educational groups with initially rising and then falling mean values from survey wave three onward. In each wave of the survey, individuals with low educational status are significantly less likely to engage in preventive behaviours than individuals with high educational status (*p* < 0.05). A significant non-linear trend with an indication of a U-shape trend can be identified in risk perception for all education groups. Raw mean values in risk perception fell sharply after the baseline survey and slightly increased at survey wave four. Over the entire observation period, persons with a low educational status had a significantly lower risk perception than persons with a high educational status (*p* ≤ 0.001). For raw mean values in perceived effectiveness of COVID-19 containment measures, we observed a significant steady decline over the course of the study across all educational groups. This trend was strongest for groups with intermediate educational status and resulted in significantly lower perceived effectiveness compared to low educational status groups in survey wave three (*p* = 0.006) and four (*p* = 0.001). Raw mean values in trust in persons and institutions handling the COVID-19 pandemic also significantly declined across all educational groups. Over the course of the study, raw mean values in trust did not significantly vary by educational status.

Figure 2 shows the marginal mean levels in preventive behaviour, risk perception, perceived effectiveness, and trust by educational status adjusted for sex, age, residence, nationality, children under 16 living in household, family status, household size, the Big Five Inventory, and income class (the full results are available in Supplementary Tables 7,8,9,10). Results shown in Fig. 2 did not substantially change after adjusting for the control variables compared to the results in Table 2. The different trend courses in preventive behaviour, risk perception, perceived effectiveness, and trust proved to be significant (*p* < 0.05) in all educational groups. Significant differences (*p* < 0.05) in preventive behaviour emerge in all survey waves to the disadvantage of low educational status groups. These educational differences were significant for risk perception from wave two onward and for trust only in wave four. Perceived effectiveness significantly varied by educational status only in survey wave four with significantly lower perceptions of effectiveness among intermediate educational groups compared to low educational groups.

Table 3 and Table 4 present the results for the multivariate analysis of trends in absolute and relative educational disparities in preventive behaviour, risk perception, perceived effectiveness, and trust (the full results are available in Supplementary Table 11,12,13,14,15,16,17,18). In all model specifications, people with the highest educational status show significantly higher preventive behaviour in comparison to the lowest educational status group not only in absolute terms but also in relative terms, as a percentage of the average in the population. Trends in SII were not significant indicating that absolute disparities in preventive behaviour did not vary over time (p > 0.05), whereas we noted a small and significant downward trend in RIIs in preventive behaviour (*p* < 0.05). For risk perception, we observed significant disparities by educational status in absolute and relative term that were not influenced by the control variables. Moreover, the non-significant trends for the SII and RII indicated that the absolute und relative disparities in risk perception did not vary over time. Finally, the results reveal consistent and non-significant absolute and relative disparities by educational status over time for perceived effectiveness and trust.

## Discussion

The one-year longitudinal study found various trends and educational disparities in preventive behaviour, risk perception, perceived effectiveness, and trust. We observed an initially rising and then falling trend in preventive behaviour with consistent absolute and relative disparities with a lower preventive behaviour among low educated individuals. Indication of a U-shaped trend with consistent significantly lower values among lower educated individuals was found for risk perception, whereas perceived effectiveness and trust decreased significantly over time but did not vary by educational status.

The general trends in preventive behaviour, risk perception, perceived effectiveness, and trust coincide with the general pandemic pattern and the measures introduced to contain the COVID-19 pandemic as shown in Fig. 1. The initially increasing and subsequently decreasing general preventive behaviour can be explained on the one hand by the introduction of more restrictive containment measures, such as the obligation to wear a protective mask, and on the other hand by the low SARS-CoV-2 incidence between May and August 2020. This mild course of the pandemic, which gained pace again from September 2020 (Fig. 1), is possibly responsible for the indicated U-sharp trend in risk perception and illustrates the sensitivity of the respondents to infection patterns during the first year of the COVID-19 pandemic in Germany. Respondents appear to react far more sensitive to the perceived effectiveness of COVID-19 containment measures and trust in individuals and institutions handling the COVID-19 pandemic. Both the perceived effectiveness and trust decreased significantly in the first year of the COVID-19 pandemic in Germany, and this despite again increasing infection rates in the last wave of observation in August/October 2020 (Fig. 1).

The relatively high level of perceived effectiveness and trust at the beginning of the pandemic in March 2020 might be explained by an anxiety effect and a cognitive need for security [30, 31]. Accordingly, the uncertainty and severity of the pandemic might lead to the so-called 'rally round the flag' effect, which results in short-term support for the entire government or political leaders of a country by the population. As the intensity of the pandemic decreased from May 2020 onwards, as evidenced by the falling numbers of infections, emotional responses might again be replaced by rational evaluation of policies and measures indicated by decreases in perceived effectiveness and trust levels. According to Smith, in the context of pandemics, risk perception in particular influences public support for people and institutions handling the pandemic and evaluation of policy measures [32]. Risk perception is influenced by a variety of social and individual factors, such as age and gender [33], professional knowledge [34], individual impact of the pandemic and the implemented measures [33], media coverage [35] or the political coordination and communication of information [36], and can therefore deviate significantly from a political-scientific risk perception [37]. One indication of difference in risk perception might be that during the first year of the COVID-19 pandemic in Germany, the restrictiveness of COVID-19 containment measures was only slightly reduced despite a mild progression of the pandemic, while the risk perception, perceived effectiveness, and trust of respondents declined sharply. This difference in risk perception must therefore be more fully considered and aligned with the needs of different social groups when communicating political decisions regarding containment measures [38].

The finding that, contrary to other studies [19–21], both trust and perceived effectiveness did not differ by educational status might indicate how extensive and across all social strata the uncertainty regarding COVID-19 containing measures and authorities handling COVID-19 was during the mild course of the pandemic in Germany. A recent study for Germany showed that moderate opponents and supporters of COVID-19 containment measures overlapped in their criticism of anxiety-inducing media coverage and fuzzy governmental communication, which might explain the general downward trend in trust and perceived effectiveness observed in our study [39]. Wegwarth et al. further indicate that the political communication of pandemic threat scenarios was devoid of uncertainty in Germany, although the communication of uncertainties seems to be particularly effective in increasing compliance of those who are currently sceptical towards COVID-19 containment measures [40]. Our findings of consistent educational disparities in preventive behaviour and risk perception contradicts with a previous trend study for Germany that only found small and varying disparities in both outcomes during the first year of the pandemic; however, methodological differences in operationalizing educational status limit the comparability of the results [19]. We consider the stability of educational disparities in preventive behaviour and risk perception as an indication of the deep underlying disparities in health and health behaviours that were already existent before the COVID-19 pandemic in Germany [41]. These socially structured disparities and its underlying factors might interact with the COVID-19 pandemic, which is why the pandemic can also be understood as a kind of magnifying glass on societal structures and conditions associated with health and health behaviours [42]. In addition to a number of different material and psychosocial factors, such as inadequate financial resources or low self-efficacy, low health literacy in particular may explain lower levels of preventive behaviour and risk perception among individuals with a low educational status [43]. COVID-19-related studies found associations of educational status with preventive behaviours and COVID-19-related health literacy such as accessing and understanding of COVID-19-related information [44, 45]. Moreover, a recent trend study for Germany showed constant disparities in knowledge of COVID-19-related information by educational status over the course of the first year of the COVID-19 pandemic [19]. A further contribution factor to the consistent educational disparities in preventive behaviours and risk perceptions might be an inadequate communication by policymakers about the risks of a SARS-CoV-2 infection and the benefits of COVID-19 containment measures. In order to reach individuals with a low educational status as well as other social groups, planned risk management by leadership in times of pandemic is necessary [38].

### Strengths and limitations

Strengths of the study include its large sample of adults, consistency in sampling and measurement over the first year of the pandemic in Germany that allow detailed descriptions of trends and differentiated analyses. Also, the use of the RII and SII provided a more complete account of trends in educational disparities in preventive behaviour, risk perception, perceived effectiveness, and trust than that either could index alone.

Our study also has limitations. First, some of the used outcomes may depend on individuals’ health status, which has not been surveyed in the GESIS Panel [18]. The results may therefore be slightly biased, as risk perception and health behaviour in particular may be influenced by individual health risk. Furthermore, in the GESIS Panel, the measurement of the perceived effectiveness of the COVID-19 containment measures referred exclusively to restrictive measures, such as the closure of recreational facilities or stores and curfews. Because these measures involve significant restrictions on individual lives, results could vary when the effectiveness of less restrictive measures, such as wearing a protective mask, is surveyed. The comparison between the first and subsequent surveys may be slightly biased because only online panellists were invited to participate in the first survey. Finally, the representativeness of the results for Germany is slightly limited because no design weight was available for the first survey wave. Sensitivity analyses of the other waves, however, have shown that the descriptive results only slightly change when a design weight was applied.

## Conclusions

This study highlights the relevance of a comprehensive and strategic management in communicating the risks of the pandemic and the benefits of COVID-19 containment measures by politics and public health. Risk and benefit communication must be adapted to the different needs of social groups in order to overcome disparities in preventive behaviour and risk perception by educational status. Further trend studies need to show whether and to what extent the trends in disparities in preventive behaviour, risk perception, perceived effectiveness, and trust by educational status change as pandemic severity increases.

## Supplementary Information

**Additional file 1: Table 1.** Panel Study Description. **Table 2.** Unit-non response rate and total response rate per survey wave. **Table 3.** Question wording, mean level and internal consistency of items measuring preventive behaviour. **Table 4.** Question wording, mean level and internal consistency of items measuring risk perception. **Table 5.** Question wording, mean level and internal consistency of items measuring perceived effectiveness of containing measures. **Table 6.** Question wording, mean level and internal consistency of items measuring trust in people and institutions. **Table 7. **Linear regression of risk perception for each survey wave separately. **Table 8.** Linear regression of risk perception for each survey wave separately. **Table 9. **Linear regression of perceived effectiveness for each survey wave separately. **Table 10.** Linear regression of trust for each survey wave separately. **Table 11.** Random effects model for absolute educational inequalities (SII) in preventive behaviours (Number of observations: 15,902; number of individuals: 4,690). **Table 12.** Random effects model for absolute educational inequalities (SII) in risk perception (Number of observations: 15,902; number of individuals: 4,690). **Table 13.** Random effects model for absolute educational inequalities (SII) in perceived effectiveness (Number of observations: 15,902; number of individuals: 4,690). **Table 14.** Random effects model for absolute educational inequalities (SII) in trust (Number of observations: 15,902; number of individuals: 4,690). **Table 15.** Random effects model for relative educational inequalities (RII) in preventive behaviours (Number of observations: 15,902; number of individuals: 4,690). **Table 16.** Random effects model for relative educational inequalities (RII) in risk perception (Number of observations: 15,902; number of individuals: 4,690). **Table 17.** Random effects model for relative educational inequalities (RII) in perceived effectiveness (Number of observations: 15,902; number of individuals: 4,690). **Table 18.** Random effects model for relative educational inequalities (RII) in trust (Number of observations: 15,902; number of individuals: 4,690).

## Data Availability

All data are available for scientific use on the website: https://www.gesis.org/gesis-panel/gesis-panel-home. The analytic scripts in Stata are available from the corresponding author on reasonable request.

## Abbreviations

ISCED: International Standard Classification of Education
CI: Confidence Interval
RII: Relative Index of Inequality
SII: Slope Index of Inequality
