# Supplementary material for: Trends in educational disparities in preventive behaviours, risk perception, perceived effectiveness and trust in the first year of the COVID-19 pandemic in Germany
Source: BMC Public Health. 2022 May 6;22:903. doi: 10.1186/s12889-022-13341-3 (PMC9073434; doi:10.1186/s12889-022-13341-3)
Supplement: Supplementary file 1 — Additional file 1: Table 1. Panel Study Description. Table 2. Unit-non response rate and total response rate per survey wave. Table 3. Question wording, mean level and internal consistency of items measuring preventive behaviour. Table 4. Question wording, mean level and internal consistency of items measuring risk perception. Table 5. Question wording, mean level and internal consistency of items measuring perceived effectiveness of containing measures. Table 6. Question wording, mean level and internal consistency of items measuring trust in people and institutions. Table 7. Linear regression of risk perception for each survey wave separately. Table 8. Linear regression of risk perception for each survey wave separately. Table 9. Linear regression of perceived effectiveness for each survey wave separately. Table 10. Linear regression of trust for each survey wave separately. Table 11. Random effects model for absolute educational inequalities (SII) in preventive behaviours (Number of observations: 15,902; number of individuals: 4,690). Table 12. Random effects model for absolute educational inequalities (SII) in risk perception (Number of observations: 15,902; number of individuals: 4,690). Table 13. Random effects model for absolute educational inequalities (SII) in perceived effectiveness (Number of observations: 15,902; number of individuals: 4,690). Table 14. Random effects model for absolute educational inequalities (SII) in trust (Number of observations: 15,902; number of individuals: 4,690). Table 15. Random effects model for relative educational inequalities (RII) in preventive behaviours (Number of observations: 15,902; number of individuals: 4,690). Table 16. Random effects model for relative educational inequalities (RII) in risk perception (Number of observations: 15,902; number of individuals: 4,690). Table 17. Random effects model for relative educational inequalities (RII) in perceived effectiveness (Number of observations: 15,902; number of individ [file 12889_2022_13341_MOESM1_ESM.docx]

**Table 1** Panel Study Description

|  |  | **Baseline survey**  (March 2020) | | **Survey wave 2**  (May/June) | | **Survey wave 3** (July/August) | | **Survey wave 4** (August/October) | |
| --- | --- | --- | --- | --- | --- | --- | --- | --- | --- |
|  |  | n | % | n | % | n | % | n | % |
| *Total* | | *3,765* | *100* | *5,123* | *100.0* | *5,063* | *100.0* | *5,001* | *100.0* |
| Cohort | |  |  |  |  |  |  |  |  |
| First cohort | | N/A | | 2,710 | 52.9 | 2,685 | 53.0 | 2,662 | 53.2 |
| Second cohort (refresher in 2016) | | N/A | | 1,122 | 21.9 | 1,112 | 22.0 | 1,101 | 22.0 |
| Third cohort (refresher in 2018) | | N/A | | 1,393 | 27.2 | 1,266 | 25.0 | 1,238 | 24.8 |
| Modus of invitation | |  |  |  |  |  |  |  |  |
| Online | | 3,765 | 100.0 | 3,730 | 72.8 | 3,686 | 72.8 | 3,647 | 72.9 |
| Offline | | 0 | 0 | 1,393 | 27.2 | 1,377 | 27.2 | 1,354 | 27.1 |
| Response rate | |  |  |  |  |  |  |  |  |
| Unit non-response | | 527 | 14.0 | 423 | 8.3 | 516 | 10.2 | 483 | 9.7 |
| Participation rate | | 3,238 | 86.0 | 4,700 | 91.7 | 4,547 | 89.8 | 4,518 | 90.3 |
| Notes: N/A: no information available. | | | | | | | | | |

| **Table 2** Unit-non response rate and total response rate per survey wave | | | | |
| --- | --- | --- | --- | --- |
| **Survey wave** | **Baseline survey** | **Survey wave 2** | **Survey wave 3** | **Survey wave 4** |
|  | (March 2020) | (May/June 2020) | (July/August 2020) | (August/October 2020) |
| *Total, % (n)* | *100.0 (3,238)* | *100.0 (4,700)* | *100.0 (4,547)* | *100.0 (4,518)* |
| Total of unit-item non-response, % (n) | 6.9 (222) | 6.9 (326) | 6.0 (274) | 6.2 (279) |
| Total of analytical sample, % (n) | 93.1 (3,016) | 93.1 (4,374) | 94.0 (4,273) | 93.8 (4,239) |
| Sex, % (n) |  |  |  |  |
| Male | 51.5 (1,552) | 49.5 (2,327) | 49.5 (2,252) | 49.6 (2,241) |
| Female | 48.5 (1,464) | 50.5 (2,372) | 50.5 (2,294) | 50.4 (2,276) |
| Total non-response, % (n) | 0.0 (1) | 0.0 (1) | 0.0 (1) | 0.0 (1) |
| Age, mean±SD | 53.9±14.0 | 56.1±14.4 | 56.3±14.3 | 56.2±14.3 |
| Unit-item non-response, % (n) | - | 0.0 (1) | - | - |
| Region, % (n) |  |  |  |  |
| West Germany | 77.0 (2,493) | 74.4 (3,495) | 74.1 (3,369) | 47.3 (3,357) |
| East Germany | 22.9 (743) | 25.5 (1,198) | 25.7 (1,170) | 25.5 (1,151) |
| Unit-item non-response, % (n) | 0.1 (2) | 0.1 (7) | 0.2 (8) | 0.2 (10) |
| Nationality, % (n) |  |  |  |  |
| German | 96.6 (3,139) | 97.1 (4,564) | 97.3 (4,424) | 97.2 (4,390) |
| Others | 2.6 (85) | 2.5 (118) | 2.5 (114) | 2.5 (113) |
| Unit-item non-response, % (n) | 0.4 (14) | 0.4 (18) | 0.2 (9) | 0.3 (15) |
| Children under 16 living  in household, % (n) |  |  |  |  |
| No | 74.1 (2,399) | 77.9 (3,660) | 78.0 (3,548) | 77.8 (3,514) |
| Yes | 25.9 (839) | 22.1 (1,040) | 22.0 (999) | 22.2 (1,004) |
| Unit-item non-response, % (n) | - | - | - | - |
| Family status, % (n) |  |  |  |  |
| Single | 23.3 (754) | 21.1 (990) | 20.6 (935) | 20.6 (930) |
| Married | 63.5 (2,056) | 62.9 (2,956) | 63.4 (2,883) | 63.4 (2,863) |
| Divorced/widowed | 13.2 (428) | 16.0 (753) | 16.0 (728) | 16.0 (724) |
| Unit-item non-response, % (n) | - | 0.0 (1) | 0.0 (1) | 0.0 (1) |
| Household size, % (n) |  |  |  |  |
| 1-person | 11.0 (358) | 13.1 (621) | 13.0 (591) | 12.7 (575) |
| 2-persons | 49.6 (1,606) | 52.3 (2,289) | 51.9 (2,360) | 51.9 (2,346) |
| 3-persons | 17.9 (579) | 15.9 (696) | 16.1 (732) | 16.3 (736) |
| 4 or more persons | 21.5 (695) | 18.7 (816) | 19.0 (864) | 19.1 (861) |
| Unit-item non-response, % (n) | - | 0.0 (1) | - | - |
| Big Five Inventory |  |  |  |  |
| Extraversion mean±SD | 2.7±0.9 | 2.7±0.9 | 2.7±0.9 | 2.7±0.9 |
| Unit-item non-response, % (n) | 0.5 (17) | 0.3 (13) | 0.3 (13) | 0.1 (4) |
| Agreeableness, mean±SD | 2.7±0.7 | 2.7±0.7 | 2.7±0.7 | 2.7±0.7 |
| Unit-item non-response, % (n) | 0.5 (17) | 0.3 (13) | 0.3 (13) | 0.1 (4) |
| Conscientiousness, mean±SD | 3.4±0.7 | 3.4±0.7 | 3.4±0.7 | 3.4±0.7 |
| Unit-item non-response, % (n) | 0.5 (17) | 0.3 (13) | 0.3 (13) | 0.1 (4) |
| Neuroticism, mean±SD | 2.3±0.8 | 2.3±0.8 | 2.3±0.8 | 2.3±0.8 |
| Unit-item non-response, % (n) | 0.5 (17) | 0.3 (13) | 0.3 (13) | 0.1 (4) |
| Open-Mindedness, mean±SD | 2.9±0.9 | 2.9±0.9 | 2.9±0.9 | 2.9±0.9 |
| Unit-item non-response, % (n) | 0.6 (18) | 0.3 (15) | 0.3 (14) | 0.1 (6) |
| Income, % (n) |  |  |  |  |
| Low | 23.0 (743) | 30.0 (1,361) | 29.2 (1,327) | 28.9 (1,304) |
| Intermediate | 37.1 (1,200) | 36.3 (1,704) | 36.2 (1,648) | 36.0 (1,628) |
| High | 36.1 (1,171) | 30.5 (1,436) | 30.5 (1,387) | 31.2 (1,409) |
| Unit-item non-response, % (n) | 3.8 (124) | 4.2 (199) | 4.1 (185) | 3.9 (177) |
| Educational status, % (n) |  |  |  |  |
| Low | 10.9 (354) | 17.9 (841) | 17.9 (813) | 15.5 (792) |
| Intermediate | 31.1 (1,006) | 33.2 (1,562) | 33.6 (1,528) | 33.4 (1,507) |
| High | 58.0 (1,878) | 48.9 (2,297) | 48.5 (2,206) | 49.1 (2,219) |
| Unit-item non-response, % (n) | - | - | - |  |
| Preventive behaviour, mean±SD | 51.1±16.3 | 62.1±17.7 | 56.1±18.2 | 54.7±18.6 |
| Unit-item non-response, % (n) | 1.6 (52) | 0.4 (18) | 0.6 (28) | 0.7 (30) |
| Risk perception, mean±SD | 50.5±17.2 | 38.9±17.7 | 37.5±16.4 | 38.5±16.8 |
| Unit-item non-response, % (n) | 1.7 (55) | 1.5 (73) | 1.3 (61) | 1.8 (82) |
| Perceived effectiveness mean±SD | 79.1±16.9 | 63.4±19.4 | 58.8±19.7 | 49.2±19.8 |
| Unit-item non-response, % (n) | 1.8 (59) | 1.1 (54) | 1.0 (43) | 1.2 (56) |
| Trust, mean±SD | 72.6±17.2 | 67.9±19.0 | 68.2±19.4 | 65.5±19.4 |
| Unit-item non-response, % (n) | 2.4 (78) | 1.5 (73) | 1.1 (52) | 1.1 (55) |

| **Table 3** Question wording, mean level and internal consistency of items measuring preventive behaviour | | | | | | | | | |
| --- | --- | --- | --- | --- | --- | --- | --- | --- | --- |
| Item | Question wording  *Which of these measures have you taken in the last 7 days?* | **Baseline survey**  (March 2020) | | **Survey wave 2**  (May/June) | | **Survey wave 3** (July/August) | | **Survey wave 4** (August/October) | |
|  |  | Mean | Kudar-Richardson 20 | Mean | Kudar-Richardson 20 | Mean | Kudar-Richardson 20 | Mean | Kudar-Richardson 20 |
| (1) | I avoided certain (busy) places. | 0.85 | 0.52 | 0.82 | 0.62 | 0.69 | 0.63 | 0.73 | 0.63 |
| (2) | I kept my distance from other people (at least 1.5 meters). | 0.81 |  | 0.93 |  | 0.89 |  | 0.89 |  |
| (3) | I have adjusted my school or work situation | 0.46 |  | 0.52 |  | 0.39 |  | 0.34 |  |
| (4) | I have quarantined myself because of symptoms. | 0.02 |  | 0.01 |  | 0.01 |  | 0.01 |  |
| (5) | I have quarantined myself even though I have no symptoms. | 0.09 |  | 0.06 |  | 0.02 |  | 0.02 |  |
| (6) | I washed my hands more often and longer. | 0.91 |  | 0.85 |  | 0.79 |  | 0.78 |  |
| (7) | I have used disinfectant. | 0.60 |  | 0.75 |  | 0.75 |  | 0.76 |  |
| (8) | I have reduced personal meetings and contacts. | 0.86 |  | 0.81 |  | 0.58 |  | 0.51 |  |
| (9) | I have worn face masks/mouth guards. | 0.04 |  | 0.95 |  | 0.95 |  | 0.95 |  |
| Notes: answer categories: 0 = no, 1 = yes. | | | | | | | | | |

| **Table 4** Question wording, mean level and internal consistency of items measuring risk perception | | | | | | | | | |
| --- | --- | --- | --- | --- | --- | --- | --- | --- | --- |
| Item | Question wording *How likely do you think it is that in the next two months ...* | **Baseline survey**  (March 2020) | | **Survey wave 2**  (May/June) | | **Survey wave 3** (July/August) | | **Survey wave 4** (August/October) | |
|  |  | Mean | Cronbachs α | Mean | Cronbachs α | Mean | Cronbachs α | Mean | Cronbachs α |
| (1) | … you will be infected with the coronavirus? | 4.09 | 0.82 | 3.30 | 0.87 | 3.14 | 0.87 | 3.20 | 0.87 |
| (2) | … someone in their immediate environment (family, friends, colleagues) will be infected with the coronavirus? | 4.55 |  | 3.62 |  | 3.46 |  | 3.54 |  |
| (3) | ... you have to be hospitalised if you become infected with the coronavirus? | 3.28 |  | 3.13 |  | 3.16 |  | 3.18 |  |
| (4) | ... you will be quarantined because of the coronavirus, regardless of whether you are infected or not? | 4.33 |  | 3.52 |  | 3.50 |  | 3.60 |  |
| (5) | ... you become infected with the coronavirus and infect other people? | 3.90 |  | 3.29 |  | 3.16 |  | 3.18 |  |
| Notes: answer categories: 1 = not at all likely, 2 = very unlikely, 3 = rather unlikely, 4 = moderately likely, 5 = rather likely, 6 = very likely, 7 = absolutely likely. | | | | | | | | | |

| **Table 5** Question wording, mean level and internal consistency of items measuring perceived effectiveness of containing measures | | | | | | | | | |
| --- | --- | --- | --- | --- | --- | --- | --- | --- | --- |
| Item | Question wording  *How effective do you think are the following policies in combating the further spread of coronavirus?* | **Baseline survey**  (March 2020) | | **Survey wave 2**  (May/June) | | **Survey wave 3** (July/August) | | **Survey wave 4** (August/October) | |
|  |  | Mean | Cronbachs α | Mean | Cronbachs α | Mean | Cronbachs α | Mean | Cronbachs α |
| (1) | Closure of day-care centres, kindergartens and schools | 4.29 | 0.87 | 3.51 | 0.85 | 3.30 | 0.87 | 2.85 | 0.87 |
| (2) | Closure of sports clubs and fitness centres | 4.38 |  | 3.88 |  | 3.70 |  | 3.32 |  |
| (3) | Closure of bars, cafes and restaurants | 4.38 |  | 3.97 |  | 3.81 |  | 3.39 |  |
| (4) | Closure of all stores except supermarkets and pharmacies | 4.06 |  | 3.45 |  | 3.23 |  | 2.72 |  |
| (5) | Ban on visiting hospitals, nursing and old people's homes | 4.45 |  | 3.94 |  | 3.71 |  | 3.38 |  |
| (6) | Curfew for anyone age 70 or older or with health problems (except for basic purchases and urgent medical care) | 3.91 |  | 3.09 |  | 2.88 |  | 2.61 |  |
| (7) | Curfew for anyone not working in health care or other critical occupations (except for basic shopping and urgent medical care) | 3.70 |  | 2.84 |  | 2.82 |  | 2.51 |  |
| Notes: answer categories: 1 = not effective at all, 2 = little effective, 3 = partly/partly, 4 = quite effective, 5 = very effective. | | | | | | | | | |

| **Table 6** Question wording, mean level and internal consistency of items measuring trust in people and institutions | | | | | | | | | |
| --- | --- | --- | --- | --- | --- | --- | --- | --- | --- |
| Item | Question wording  *How much do they trust the following people and institutions*  *to handle coronavirus?* | **Baseline survey**  (March 2020) | | **Survey wave 2**  (May/June) | | **Survey wave 3** (July/August) | | **Survey wave 4** (August/October) | |
|  |  | Mean | Cronbachs α | Mean | Cronbachs α | Mean | Cronbachs α | Mean | Cronbachs α |
| (1) | Your general practitioner | 4.15 | 0.89 | 4.16 | 0.91 | 4.14 | 0.91 | 4.06 | 0.91 |
| (2) | Local health authority | 3.81 |  | 3.64 |  | 3.65 |  | 3.50 |  |
| (3) | Municipal and city administration | 3.44 |  | 3.33 |  | 3.33 |  | 3.19 |  |
| (4) | Robert Koch Institute (RKI) | 4.44 |  | 3.93 |  | 3.98 |  | 3.88 |  |
| (5) | Federal government | 3.66 |  | 3.54 |  | 3.60 |  | 3.45 |  |
| (6) | Chancellor | 3.57 |  | 3.65 |  | 3.67 |  | 3.55 |  |
| (7) | Ministry of Health | 3.82 |  | 3.62 |  | 3.67 |  | 3.51 |  |
| (8) | World Health Organization (WHO) | 3.96 |  | 3.47 |  | 3.49 |  | 3.38 |  |
| (9) | Scientists | 4.24 |  | 3.99 |  | 3.99 |  | 3.92 |  |
| Notes: answer categories: 1 = do not trust at all, 2 = rather not trust, 3 = neither, 4 = rather trust, 5 = trust completely. | | | | | | | | | |

| **Table 7** Linear regression of risk perception for each survey wave separately | | | | |
| --- | --- | --- | --- | --- |
|  | **Baseline survey** | **Survey wave 2** | **Survey wave 3** | **Survey wave 4** |
|  | (March 2020) | (May/June 2020) | (July/August 2020) | (August/October 2020) |
| Educational status (ref.: Low) |  |  |  |  |
| Intermediate | 1.376 | 2.619^**^ | 2.049^**^ | 1.225 |
|  | (-0.778,3.531) | (1.030,4.208) | (0.558,3.540) | (-0.321,2.771) |
| High | 1.963 | 3.811^***^ | 3.121^***^ | 2.640^**^ |
|  | (-0.164,4.089) | (2.192,5.431) | (1.594,4.648) | (1.063,4.216) |
| Gender (ref.: Male) |  |  |  |  |
| Female | -0.440 | 0.694 | 1.189^*^ | 1.923^***^ |
|  | (-1.721,0.841) | (-0.405,1.793) | (0.156,2.221) | (0.856,2.990) |
| Age | -0.263^***^ | -0.034 | -0.017 | -0.012 |
|  | (-0.323,-0.203) | (-0.085,0.018) | (-0.066,0.031) | (-0.062,0.038) |
| Region (ref.: West-Germany) |  |  |  |  |
| East-Germany | -1.974^**^ | -1.963^**^ | -2.174^***^ | -2.593^***^ |
|  | (-3.424,-0.524) | (-3.186,-0.739) | (-3.320,-1.029) | (-3.776,-1.410) |
| Nationality (ref.: German) |  |  |  |  |
| Others | -3.254 | -4.190^*^ | -0.056 | 1.677 |
|  | (-7.117,0.609) | (-7.602,-0.778) | (-3.293,3.180) | (-1.593,4.947) |
| Children under 16 living in household (ref.: No) |  |  |  |  |
| Yes | -0.152 | -0.568 | -0.430 | -1.543 |
|  | (-2.165,1.861) | (-2.353,1.217) | (-2.118,1.258) | (-3.259,0.173) |
| Family status (ref.: Single) |  |  |  |  |
| Married | 0.980 | 1.478 | 1.851^*^ | 0.431 |
|  | (-0.995,2.956) | (-0.264,3.220) | (0.200,3.501) | (-1.256,2.119) |
| Divorced/widowed | 0.831 | 0.310 | 0.884 | -0.386 |
|  | (-1.534,3.196) | (-1.675,2.294) | (-0.992,2.761) | (-2.303,1.530) |
| Household size (ref.: 1 person) |  |  |  |  |
| 2 persons | -0.126 | 0.444 | 0.048 | 0.578 |
|  | (-2.394,2.141) | (-1.406,2.294) | (-1.699,1.794) | (-1.234,2.390) |
| 3 persons | -0.231 | -0.071 | -0.359 | 1.022 |
|  | (-2.946,2.483) | (-2.367,2.226) | (-2.515,1.797) | (-1.216,3.259) |
| 4 persons | 0.307 | 1.958 | 1.420 | 3.309^**^ |
|  | (-2.737,3.351) | (-0.622,4.538) | (-1.019,3.859) | (0.801,5.818) |
| Big Five Inventory |  |  |  |  |
| Extraversion | 0.097 | -0.236 | -0.276 | 0.021 |
|  | (-0.625,0.818) | (-0.867,0.394) | (-0.871,0.319) | (-0.589,0.632) |
| Agreeableness | -0.777 | -0.979^*^ | -0.475 | -0.964^**^ |
|  | (-1.665,0.111) | (-1.735,-0.222) | (-1.187,0.238) | (-1.694,-0.233) |
| Conscientiousness | -0.023 | -1.340^**^ | -1.051^**^ | -1.068^**^ |
|  | (-0.943,0.897) | (-2.138,-0.541) | (-1.806,-0.295) | (-1.843,-0.293) |
| Neuroticism | 0.952^*^ | 1.117^**^ | 1.139^***^ | 1.247^***^ |
|  | (0.169,1.734) | (0.443,1.792) | (0.504,1.774) | (0.594,1.900) |
| Open-Mindedness | 0.566 | 1.105^***^ | 0.931^**^ | 0.886^**^ |
|  | (-0.141,1.273) | (0.493,1.716) | (0.356,1.506) | (0.291,1.481) |
| Income (ref.: Low) |  |  |  |  |
| Moderate | 2.548^**^ | 1.287 | 1.098 | 1.975^**^ |
|  | (0.868,4.228) | (-0.089,2.663) | (-0.199,2.394) | (0.640,3.311) |
| High | 3.529^***^ | 0.830 | 0.698 | 1.425 |
|  | (1.687,5.370) | (-0.751,2.410) | (-0.789,2.185) | (-0.105,2.956) |
| Intercept | 58.98^***^ | 38.32^***^ | 34.52^***^ | 35.80^***^ |
|  | (52.896,65.070) | (33.089,43.555) | (29.594,39.450) | (30.752,40.843) |
| N | 3,016 | 4,374 | 4,273 | 4,239 |
| R^2^ | 0.0656 | 0.0269 | 0.0263 | 0.0343 |
| Notes: ^*^ *p* < 0.05 - ^**^ *p* < 0.01 - ^***^ *p* < 0.001 | | | | |

| **Table 8** Linear regression of risk perception for each survey wave separately | | | | |
| --- | --- | --- | --- | --- |
|  | **Baseline survey** | **Survey wave 2** | **Survey wave 3** | **Survey wave 4** |
|  | (March 2020) | (May/June 2020) | (July/August 2020) | (August/October 2020) |
| Educational status (ref.: Low) |  |  |  |  |
| Intermediate | 1.376 | 2.619^**^ | 2.049^**^ | 1.225 |
|  | (-0.778,3.531) | (1.030,4.208) | (0.558,3.540) | (-0.321,2.771) |
| High | 1.963 | 3.811^***^ | 3.121^***^ | 2.640^**^ |
|  | (-0.164,4.089) | (2.192,5.431) | (1.594,4.648) | (1.063,4.216) |
| Gender (ref.: Male) |  |  |  |  |
| Female | -0.440 | 0.694 | 1.189^*^ | 1.923^***^ |
|  | (-1.721,0.841) | (-0.405,1.793) | (0.156,2.221) | (0.856,2.990) |
| Age | -0.263^***^ | -0.034 | -0.017 | -0.012 |
|  | (-0.323,-0.203) | (-0.085,0.018) | (-0.066,0.031) | (-0.062,0.038) |
| Region (ref.: West-Germany) |  |  |  |  |
| East-Germany | -1.974^**^ | -1.963^**^ | -2.174^***^ | -2.593^***^ |
|  | (-3.424,-0.524) | (-3.186,-0.739) | (-3.320,-1.029) | (-3.776,-1.410) |
| Nationality (ref.: German) |  |  |  |  |
| Others | -3.254 | -4.190^*^ | -0.056 | 1.677 |
|  | (-7.117,0.609) | (-7.602,-0.778) | (-3.293,3.180) | (-1.593,4.947) |
| Children under 16 living in household (ref.: No) |  |  |  |  |
| Yes | -0.152 | -0.568 | -0.430 | -1.543 |
|  | (-2.165,1.861) | (-2.353,1.217) | (-2.118,1.258) | (-3.259,0.173) |
| Family status (ref.: Single) |  |  |  |  |
| Married | 0.980 | 1.478 | 1.851^*^ | 0.431 |
|  | (-0.995,2.956) | (-0.264,3.220) | (0.200,3.501) | (-1.256,2.119) |
| Divorced/widowed | 0.831 | 0.310 | 0.884 | -0.386 |
|  | (-1.534,3.196) | (-1.675,2.294) | (-0.992,2.761) | (-2.303,1.530) |
| Household size (ref.: 1 person) |  |  |  |  |
| 2 persons | -0.126 | 0.444 | 0.048 | 0.578 |
|  | (-2.394,2.141) | (-1.406,2.294) | (-1.699,1.794) | (-1.234,2.390) |
| 3 persons | -0.231 | -0.071 | -0.359 | 1.022 |
|  | (-2.946,2.483) | (-2.367,2.226) | (-2.515,1.797) | (-1.216,3.259) |
| 4 persons | 0.307 | 1.958 | 1.420 | 3.309^**^ |
|  | (-2.737,3.351) | (-0.622,4.538) | (-1.019,3.859) | (0.801,5.818) |
| Big Five Inventory |  |  |  |  |
| Extraversion | 0.097 | -0.236 | -0.276 | 0.021 |
|  | (-0.625,0.818) | (-0.867,0.394) | (-0.871,0.319) | (-0.589,0.632) |
| Agreeableness | -0.777 | -0.979^*^ | -0.475 | -0.964^**^ |
|  | (-1.665,0.111) | (-1.735,-0.222) | (-1.187,0.238) | (-1.694,-0.233) |
| Conscientiousness | -0.023 | -1.340^**^ | -1.051^**^ | -1.068^**^ |
|  | (-0.943,0.897) | (-2.138,-0.541) | (-1.806,-0.295) | (-1.843,-0.293) |
| Neuroticism | 0.952^*^ | 1.117^**^ | 1.139^***^ | 1.247^***^ |
|  | (0.169,1.734) | (0.443,1.792) | (0.504,1.774) | (0.594,1.900) |
| Open-Mindedness | 0.566 | 1.105^***^ | 0.931^**^ | 0.886^**^ |
|  | (-0.141,1.273) | (0.493,1.716) | (0.356,1.506) | (0.291,1.481) |
| Income (ref.: Low) |  |  |  |  |
| Moderate | 2.548^**^ | 1.287 | 1.098 | 1.975^**^ |
|  | (0.868,4.228) | (-0.089,2.663) | (-0.199,2.394) | (0.640,3.311) |
| High | 3.529^***^ | 0.830 | 0.698 | 1.425 |
|  | (1.687,5.370) | (-0.751,2.410) | (-0.789,2.185) | (-0.105,2.956) |
| Intercept | 58.98^***^ | 38.32^***^ | 34.52^***^ | 35.80^***^ |
|  | (52.896,65.070) | (33.089,43.555) | (29.594,39.450) | (30.752,40.843) |
| N | 3016 | 4374 | 4273 | 4239 |
| R^2^ | 0.0656 | 0.0269 | 0.0263 | 0.0343 |
| Notes: ^*^ *p* < 0.05 - ^**^ *p* < 0.01 - ^***^ *p* < 0.001 | | | | |

| **Table 9** Linear regression of perceived effectiveness for each survey wave separately | | | | |
| --- | --- | --- | --- | --- |
|  | **Baseline survey** | **Survey wave 2** | **Survey wave 3** | **Survey wave 4** |
|  | (March 2020) | (May/June 2020) | (July/August 2020) | (August/October 2020) |
| Educational status (ref.: Low) |  |  |  |  |
| Intermediate | 0.961 | -0.634 | -1.746 | -2.178^*^ |
|  | (-1.181,3.103) | (-2.399,1.131) | (-3.561,0.069) | (-4.011,-0.345) |
| High | -0.886 | 0.046 | -0.170 | -0.463 |
|  | (-3.000,1.228) | (-1.753,1.846) | (-2.029,1.689) | (-2.332,1.405) |
| Gender (ref.: Male) |  |  |  |  |
| Female | 2.923^***^ | 1.800^**^ | -0.677 | -3.035^***^ |
|  | (1.649,4.196) | (0.580,3.021) | (-1.935,0.580) | (-4.300,-1.770) |
| Age | -0.057 | 0.010 | -0.032 | -0.087^*^ |
|  | (-0.116,0.002) | (-0.056,0.075) | (-0.101,0.037) | (-0.155,-0.019) |
| Region (ref.: West-Germany) |  |  |  |  |
| East-Germany | -0.054 | 0.0583^*^ | 0.042 | -0.021 |
|  | (-0.114,0.005) | (0.001,0.115) | (-0.018,0.101) | (-0.080,0.038) |
| Nationality (ref.: German) |  |  |  |  |
| Others | 5.455^**^ | 1.925 | 1.161 | 2.882 |
|  | (1.615,9.296) | (-1.866,5.716) | (-2.779,5.101) | (-0.994,6.758) |
| Children under 16 living in household (ref.: No) |  |  |  |  |
| Yes | 1.122 | -0.872 | -1.977 | -0.782 |
|  | (-0.880,3.123) | (-2.855,1.111) | (-4.032,0.078) | (-2.816,1.252) |
| Family status (ref.: Single) |  |  |  |  |
| Married | 1.557 | 0.000 | 0.360 | -0.658 |
|  | (-0.407,3.521) | (-1.935,1.935) | (-1.649,2.369) | (-2.659,1.342) |
| Divorced/widowed | -0.060 | -1.455 | -0.300 | -1.222 |
|  | (-2.411,2.292) | (-3.660,0.750) | (-2.584,1.985) | (-3.494,1.050) |
| Household size (ref.: 1 person) |  |  |  |  |
| 2 persons | -0.395 | -1.251 | -0.385 | -0.907 |
|  | (-2.649,1.859) | (-3.307,0.804) | (-2.511,1.741) | (-3.055,1.240) |
| 3 persons | -1.244 | -1.590 | -0.479 | -1.913 |
|  | (-3.942,1.455) | (-4.142,0.961) | (-3.104,2.146) | (-4.565,0.739) |
| 4 persons | -1.752 | -1.777 | 0.072 | -1.213 |
|  | (-4.779,1.274) | (-4.643,1.090) | (-2.897,3.041) | (-4.187,1.761) |
| Big Five Inventory |  |  |  |  |
| Extraversion | 0.868^*^ | -0.398 | -0.767^*^ | -0.686 |
|  | (0.151,1.586) | (-1.099,0.302) | (-1.492,-0.043) | (-1.410,0.038) |
| Agreeableness | 1.128^*^ | 0.486 | 0.197 | -0.248 |
|  | (0.245,2.011) | (-0.355,1.326) | (-0.671,1.064) | (-1.114,0.617) |
| Conscientiousness | 2.132^***^ | 0.814 | 0.865 | -0.062 |
|  | (1.217,3.046) | (-0.073,1.701) | (-0.055,1.785) | (-0.981,0.856) |
| Neuroticism | 0.671 | 0.490 | 0.363 | 0.980^*^ |
|  | (-0.107,1.449) | (-0.259,1.240) | (-0.410,1.137) | (0.207,1.754) |
| Open-Mindedness | 0.532 | 0.822^*^ | 0.817^*^ | 0.776^*^ |
|  | (-0.171,1.235) | (0.143,1.501) | (0.116,1.517) | (0.070,1.481) |
| Income (ref.: Low) |  |  |  |  |
| Moderate | 0.943 | 0.420 | 0.909 | 0.789 |
|  | (-0.727,2.614) | (-1.109,1.949) | (-0.669,2.487) | (-0.794,2.372) |
| High | 2.680^**^ | 1.394 | 1.864^*^ | 1.918^*^ |
|  | (0.850,4.511) | (-0.362,3.150) | (0.055,3.674) | (0.104,3.732) |
| Intercept | 63.71^***^ | 54.71^***^ | 53.00^***^ | 52.71^***^ |
|  | (57.657,69.761) | (48.901,60.529) | (47.004,59.002) | (46.729,58.689) |
| N | 3016 | 4374 | 4273 | 4239 |
| R^2^ | 0.0427 | 0.0153 | 0.0131 | 0.0243 |
| Notes: ^*^ *p* < 0.05 - ^**^ *p* < 0.01 - ^***^ *p* < 0.001 | | | | |

| **Table 10** Linear regression of trust for each survey wave separately | | | | |
| --- | --- | --- | --- | --- |
|  | **Baseline survey** | **Survey wave 2** | **Survey wave 3** | **Survey wave 4** |
|  | (March 2020) | (May/June 2020) | (July/August 2020) | (August/October 2020) |
| Educational status (ref.: Low) | |  |  |  |
| Intermediate | -1.015 | -0.324 | -0.912 | -0.403 |
|  | (-3.177,1.146) | (-2.004,1.357) | (-2.655,0.832) | (-2.157,1.352) |
| High | -0.911 | 1.526 | 0.784 | 2.070* |
|  | (-3.044,1.222) | (-0.188,3.239) | (-1.002,2.570) | (0.281,3.859) |
| Gender (ref.: Male) | |  |  |  |
| Female | 2.370*** | 1.815** | 1.519* | 2.154*** |
|  | (1.085,3.655) | (0.653,2.978) | (0.311,2.726) | (0.943,3.365) |
| Age | 0.0943** | 0.178*** | 0.157*** | 0.170*** |
|  | (0.034,0.154) | (0.124,0.232) | (0.100,0.214) | (0.113,0.226) |
| Region (ref.: West-Germany) | |  |  |  |
| East-Germany | 0.328 | -1.245 | -1.718* | -2.236** |
|  | (-1.127,1.782) | (-2.539,0.049) | (-3.058,-0.379) | (-3.579,-0.894) |
| Nationality (ref.: German) |  |  |  |  |
| Others | 0.247 | -4.363* | -2.410 | -3.288 |
|  | (-3.628,4.122) | (-7.973,-0.753) | (-6.195,1.374) | (-6.999,0.422) |
| Children under 16 living in household  (ref.: No) | | |  |  |
| Yes | -0.139 | -0.356 | -0.820 | -0.259 |
|  | (-2.159,1.880) | (-2.244,1.533) | (-2.794,1.154) | (-2.207,1.688) |
| Family status (ref.: Single) | |  |  |  |
| Married | 0.823 | 0.579 | 1.077 | 0.644 |
|  | (-1.159,2.805) | (-1.264,2.422) | (-0.853,3.007) | (-1.271,2.559) |
| Divorced/widowed | -1.824 | -1.809 | -1.338 | -2.233* |
|  | (-4.196,0.549) | (-3.909,0.290) | (-3.532,0.856) | (-4.408,-0.058) |
| Household size (ref.: 1 person) | |  |  |  |
| 2 persons | -3.636** | -1.384 | -2.728** | -2.419* |
|  | (-5.910,-1.362) | (-3.341,0.574) | (-4.771,-0.686) | (-4.474,-0.363) |
| 3 persons | -3.015* | -2.354 | -3.782** | -3.239* |
|  | (-5.738,-0.292) | (-4.783,0.076) | (-6.303,-1.261) | (-5.778,-0.700) |
| 4 persons | -4.380** | -3.616** | -4.139** | -4.264** |
|  | (-7.433,-1.326) | (-6.346,-0.886) | (-6.991,-1.287) | (-7.110,-1.417) |
| Big Five Inventory | |  |  |  |
| Extraversion | 0.471 | 0.202 | 0.095 | 0.020 |
|  | (-0.253,1.195) | (-0.465,0.869) | (-0.601,0.791) | (-0.673,0.713) |
| Agreeableness | 4.068*** | 4.223*** | 3.990*** | 4.002*** |
|  | (3.177,4.959) | (3.422,5.023) | (3.157,4.823) | (3.173,4.831) |
| Conscientiousness | -0.031 | 0.518 | 0.940* | 0.379 |
|  | (-0.954,0.892) | (-0.327,1.362) | (0.056,1.823) | (-0.500,1.258) |
| Neuroticism | -0.032 | 0.299 | 0.302 | -0.134 |
|  | (-0.817,0.753) | (-0.415,1.013) | (-0.441,1.045) | (-0.875,0.606) |
| Open-Mindedness | 0.895* | 0.787* | 0.674* | 0.863* |
|  | (0.186,1.605) | (0.140,1.434) | (0.001,1.347) | (0.188,1.538) |
| Income (ref.: Low) | |  |  |  |
| Moderate | 1.838* | 0.849 | 1.631* | 1.757* |
|  | (0.153,3.523) | (-0.607,2.305) | (0.116,3.147) | (0.241,3.272) |
| High | 3.561*** | 2.928*** | 4.330*** | 4.235*** |
|  | (1.715,5.408) | (1.256,4.600) | (2.591,6.068) | (2.498,5.971) |
| Intercept | 53.33*** | 40.59*** | 42.63*** | 40.72*** |
|  | (47.227,59.440) | (35.057,46.130) | (36.873,48.397) | (34.992,46.442) |
| N | 3016 | 4374 | 4273 | 4239 |
| R^2^ | 0.0532 | 0.063 | 0.0577 | 0.0627 |
| Notes: ^*^ *p* < 0.05 - ^**^ *p* < 0.01 - ^***^ *p* < 0.001 | | | | |

| **Table 11** Random effects model for absolute educational inequalities (SII) in preventive behaviours (Number of observations: 15,902; number of individuals: 4,690) | | | | |
| --- | --- | --- | --- | --- |
|  | M1 | M2 | M3 | M4 |
|  | beta | beta | beta | beta |
|  | (95%-CI) | (95%-CI) | (95%-CI) | (95%-CI) |
| SII | 6.565** | 6.779** | 7.231** | 6.055** |
|  | (2.131,10.998) | (2.337,11.222) | (2.779,11.683) | (1.566,10.544) |
| Trend | 10.59*** | 10.52*** | 10.58*** | 10.61*** |
| (linear) | (8.960,12.230) | (8.886,12.155) | (8.942,12.208) | (8.980,12.247) |
| Trend (quadratic) | -3.343*** | -3.324*** | -3.335*** | -3.344*** |
|  | (-3.827,-2.860) | (-3.808,-2.840) | (-3.818,-2.852) | (-3.827,-2.861) |
| SII*Trend (linear) | -1.037 | -0.967 | -1.008 | -1.050 |
|  | (-3.631,1.556) | (-3.561,1.626) | (-3.599,1.583) | (-3.641,1.541) |
| SII*Trend (quadratic) | 0.316 | 0.296 | 0.303 | 0.313 |
|  | (-0.463,1.095) | (-0.483,1.075) | (-0.475,1.082) | (-0.466,1.091) |
| Big Five Inventory |  |  |  |  |
| Extraversion |  | 0.257 | 0.059 | 0.014 |
|  |  | (-0.251,0.765) | (-0.441,0.559) | (-0.485,0.514) |
| Agreeableness |  | 0.629* | 0.335 | 0.427 |
|  |  | (0.098,1.160) | (-0.199,0.868) | (-0.108,0.961) |
| Conscientiousness |  | 0.908*** | 0.867*** | 0.885*** |
|  |  | (0.416,1.400) | (0.382,1.352) | (0.400,1.369) |
| Neuroticism |  | 2.055*** | 1.912*** | 1.897*** |
|  |  | (1.426,2.684) | (1.279,2.545) | (1.265,2.528) |
| Open-Mindedness |  | 0.741* | 0.499 | 0.564 |
|  |  | (0.132,1.350) | (-0.099,1.098) | (-0.035,1.162) |
| Gender (ref.: male) |  | 0.257 |  |  |
| Female |  |  | 2.376*** | 2.530*** |
|  |  |  | (1.510,3.242) | (1.662,3.398) |
| Age |  |  | 0.028 | 0.032 |
|  |  |  | (-0.012,0.069) | (-0.008,0.073) |
| Region (ref.: wester Germany) | |  |  |  |
| Eastern Germany |  |  | -6.297*** | -6.010*** |
|  |  |  | (-7.242,-5.353) | (-6.964,-5.056) |
| Nationality (ref.: German) |  |  |  |  |
| Others |  |  | 1.105 | 1.349 |
|  |  |  | (-1.557,3.767) | (-1.312,4.010) |
| Children under 16 living in household (ref.: no) | | |  |  |
| Yes |  |  | 0.291 | 0.290 |
|  |  |  | (-1.107,1.689) | (-1.107,1.686) |
| Family status (ref.: single) | |  |  |  |
| Married |  |  | 2.132^**^ | 1.575^*^ |
|  |  |  | (0.787,3.476) | (0.205,2.945) |
| Divorced/widowed |  |  | -0.349 | -0.417 |
|  |  |  | (-1.921,1.223) | (-1.989,1.154) |
| Household size (ref.: 1 person) | |  |  |  |
| 2 persons |  |  | 1.383 | 0.933 |
|  |  |  | (-0.072,2.838) | (-0.539,2.406) |
| 3 persons |  |  | 0.744 | 0.084 |
|  |  |  | (-1.041,2.530) | (-1.728,1.897) |
| 4 persons |  |  | 1.520 | 0.840 |
|  |  |  | (-0.496,3.536) | (-1.200,2.880) |
| Income (ref.: low) |  |  |  |  |
| Moderate |  |  |  | 1.460^**^ |
|  |  |  |  | (0.372,2.547) |
| High |  |  |  | 2.544^***^ |
|  |  |  |  | (1.297,3.791) |
| Intercept | 38.55^***^ | 24.69^***^ | 23.17^***^ | 22.61^***^ |
|  | (35.726,41.373) | (20.300,29.071) | (18.292,28.056) | (17.715,27.508) |
| $R^{2}$ | 0.032 | 0.042 | 0.077 | 0.079 |
| $\hat{\sigma}_{u}$ | 13.069 | 12.928 | 12.519 | 12.494 |
| $\hat{\sigma}_{e}$ | 12.210 | 12.210 | 12.210 | 12.210 |
| $\hat{\rho}$ | 0.534 | 0.529 | 0.512 | 0.511 |
| *Notes*: M1: SII with linear and quadratic trend variable and an interaction between SII and the linear and quadratic trend variable. M2: M1+the big five inventory. M3: M2+ sex, age, region, nationality, children under 16 in household, family status, and household size. M4: M3+income class.  $\hat{\sigma}_{u}$: between-unit standard deviation. $\hat{\sigma}_{e}$: within-unit standard deviation. $\hat{\rho}$: proportion of variance explained by between-unit differences. | | | | |

| **Table 12** Random effects model for absolute educational inequalities (SII) in risk perception (Number of observations: 15,902; number of individuals: 4,690) | | | | |
| --- | --- | --- | --- | --- |
|  | M1 | M2 | M3 | M4 |
|  | beta | beta | beta | beta |
|  | (95%-CI) | (95%-CI) | (95%-CI) | (95%-CI) |
| SII | 11.23*** | 10.44*** | 9.443*** | 8.749*** |
|  | (7.046,15.420) | (6.242,14.636) | (5.222,13.663) | (4.491,13.007) |
| Trend | -10.81*** | -10.80*** | -10.72*** | -10.68*** |
| (linear) | (-12.353,-9.267) | (-12.344,-9.259) | (-12.265,-9.180) | (-12.223,-9.138) |
| Trend (quadratic) | 2.617*** | 2.615*** | 2.597*** | 2.587*** |
|  | (2.160,3.073) | (2.158,3.071) | (2.140,3.053) | (2.131,3.044) |
| SII*Trend (linear) | -2.440 | -2.432 | -2.488* | -2.543* |
|  | (-4.888,0.007) | (-4.879,0.015) | (-4.935,-0.042) | (-4.990,-0.096) |
| SII*Trend (quadratic) | 0.519 | 0.517 | 0.528 | 0.541 |
|  | (-0.217,1.254) | (-0.218,1.252) | (-0.207,1.263) | (-0.194,1.276) |
| Big Five Inventory |  |  |  |  |
| Extraversion |  | 0.161 | -0.0241 | -0.0436 |
|  |  | (-0.322,0.644) | (-0.508,0.460) | (-0.528,0.440) |
| Agreeableness |  | 1.201*** | 0.988*** | 1.060*** |
|  |  | (0.696,1.707) | (0.471,1.505) | (0.542,1.578) |
| Conscientiousness |  | 0.767** | 0.852*** | 0.865*** |
|  |  | (0.299,1.235) | (0.382,1.322) | (0.396,1.335) |
| Neuroticism |  | -1.090*** | -0.986** | -0.991** |
|  |  | (-1.689,-0.492) | (-1.599,-0.374) | (-1.603,-0.379) |
| Open-Mindedness |  | -0.840** | -0.915** | -0.865** |
|  |  | (-1.419,-0.261) | (-1.495,-0.336) | (-1.445,-0.285) |
| Gender (ref.: male) |  |  |  |  |
| Female |  |  | 2.376*** | 2.530*** |
|  |  |  | (1.510,3.242) | (1.662,3.398) |
| Age |  |  | 0.028 | 0.032 |
|  |  |  | (-0.012,0.069) | (-0.008,0.073) |
| Region (ref.: wester Germany) | |  |  |  |
| Eastern Germany |  |  | -2.208*** | -2.034*** |
|  |  |  | (-3.123,-1.293) | (-2.958,-1.109) |
| Nationality (ref.: German) |  |  |  |  |
| Others |  |  | -1.768 | -1.628 |
|  |  |  | (-4.347,0.810) | (-4.206,0.950) |
| Children under 16 living in household (ref.: no) | | |  |  |
| Yes |  |  | -0.497 | -0.538 |
|  |  |  | (-1.851,0.857) | (-1.891,0.815) |
| Family status (ref.: single) | |  |  |  |
| Married |  |  | 1.543* | 1.156 |
|  |  |  | (0.240,2.845) | (-0.172,2.484) |
| Divorced/widowed |  |  | 0.503 | 0.525 |
|  |  |  | (-1.020,2.026) | (-0.997,2.048) |
| Household size (ref.: 1 person) | |  |  |  |
| 2 persons |  |  | 0.914 | 0.505 |
|  |  |  | (-0.496,2.323) | (-0.922,1.932) |
| 3 persons |  |  | 0.667 | 0.174 |
|  |  |  | (-1.063,2.397) | (-1.582,1.931) |
| 4 persons |  |  | 2.490* | 2.010* |
|  |  |  | (0.538,4.443) | (0.034,3.987) |
| Income (ref.: low) |  |  |  |  |
| Moderate |  |  |  | 1.841*** |
|  |  |  |  | (0.787,2.894) |
| High |  |  |  | 1.669** |
|  |  |  |  | (0.461,2.877) |
| Intercept | 55.25*** | 56.25*** | 59.12*** | 58.38*** |
|  | (52.584,57.917) | (52.093,60.413) | (54.422,63.814) | (53.668,63.091) |
| $R^{2}$ | 0.080 | 0.088 | 0.098 | 0.099 |
| $\hat{\sigma}_{u}$ | 12.392 | 12.306 | 12.195 | 12.176 |
| $\hat{\sigma}_{e}$ | 11.500 | 11.500 | 11.500 | 11.500 |
| $\hat{\rho}$ | 0.54 | 0.53 | 0.53 | 0.53 |
| *Notes*: M1: SII with linear and quadratic trend variable and an interaction between SII and the linear and quadratic trend variable. M2: M1+the big five inventory. M3: M2+ sex, age, region, nationality, children under 16 in household, family status, and household size. M4: M3+income class.  $\hat{\sigma}_{u}$: between-unit standard deviation. $\hat{\sigma}_{e}$: within-unit standard deviation. $\hat{\rho}$: proportion of variance explained by between-unit differences. | | | | |

| **Table 13** Random effects model for absolute educational inequalities (SII) in perceived effectiveness (Number of observations: 15,902; number of individuals: 4,690) | | | | |
| --- | --- | --- | --- | --- |
|  | M1 | M2 | M3 | M4 |
|  | beta | beta | beta | beta |
|  | (95%-CI) | (95%-CI) | (95%-CI) | (95%-CI) |
| SII | -0.145 | -0.269 | 0.217 | -0.688 |
|  | (-5.036,4.747) | (-5.174,4.636) | (-4.716,5.150) | (-5.662,4.285) |
| Trend | -13.67*** | -13.70*** | -13.69*** | -13.67*** |
| (linear) | (-15.487,-11.858) | (-15.514,-11.885) | (-15.502,-11.874) | (-15.482,-11.853) |
| Trend (quadratic) | 1.276*** | 1.283*** | 1.281*** | 1.276*** |
|  | (0.739,1.812) | (0.746,1.819) | (0.744,1.817) | (0.739,1.813) |
| SII*Trend (linear) | 0.0764 | 0.105 | 0.101 | 0.0848 |
|  | (-2.802,2.955) | (-2.774,2.983) | (-2.777,2.980) | (-2.794,2.963) |
| SII*Trend (quadratic) | 0.291 | 0.283 | 0.283 | 0.286 |
|  | (-0.574,1.156) | (-0.582,1.148) | (-0.582,1.148) | (-0.578,1.151) |
| Big Five Inventory |  |  |  |  |
| Extraversion |  | -0.268 | -0.247 | -0.285 |
|  |  | (-0.812,0.275) | (-0.793,0.298) | (-0.831,0.261) |
| Agreeableness |  | 0.460 | 0.517 | 0.579 |
|  |  | (-0.107,1.028) | (-0.065,1.100) | (-0.005,1.164) |
| Conscientiousness |  | 0.639* | 0.640* | 0.652* |
|  |  | (0.113,1.165) | (0.110,1.169) | (0.123,1.182) |
| Neuroticism |  | 0.689* | 0.818* | 0.804* |
|  |  | (0.017,1.362) | (0.127,1.509) | (0.114,1.495) |
| Open-Mindedness |  | 0.391 | 0.350 | 0.393 |
|  |  | (-0.260,1.042) | (-0.304,1.003) | (-0.261,1.047) |
| Gender (ref.: male) |  |  |  |  |
| Female |  |  | -0.085 | 0.035 |
|  |  |  | (-1.030,0.861) | (-0.914,0.983) |
| Age |  |  | 0.022 | 0.025 |
|  |  |  | (-0.023,0.066) | (-0.020,0.069) |
| Region (ref.: wester Germany) | |  |  |  |
| Eastern Germany |  |  | -3.304*** | -3.086*** |
|  |  |  | (-4.336,-2.273) | (-4.129,-2.043) |
| Nationality (ref.: German) |  |  |  |  |
| Others |  |  | 2.436 | 2.625 |
|  |  |  | (-0.472,5.343) | (-0.284,5.534) |
| Children under 16 living in household (ref.: no) | | |  |  |
| Yes |  |  | -0.813 | -0.794 |
|  |  |  | (-2.340,0.713) | (-2.321,0.732) |
| Family status (ref.: single) | |  |  |  |
| Married |  |  | 0.366 | -0.033 |
|  |  |  | (-1.103,1.834) | (-1.531,1.465) |
| Divorced/widowed |  |  | -0.875 | -0.959 |
|  |  |  | (-2.592,0.841) | (-2.676,0.759) |
| Household size (ref.: 1 person) | |  |  |  |
| 2 persons |  |  | -0.305 | -0.580 |
|  |  |  | (-1.894,1.284) | (-2.189,1.030) |
| 3 persons |  |  | -0.700 | -1.156 |
|  |  |  | (-2.650,1.251) | (-3.137,0.825) |
| 4 persons |  |  | -0.245 | -0.729 |
|  |  |  | (-2.446,1.956) | (-2.959,1.501) |
| Income (ref.: low) |  |  |  |  |
| Moderate |  |  |  | 0.640 |
|  |  |  |  | (-0.548,1.829) |
| High |  |  |  | 1.870** |
|  |  |  |  | (0.507,3.233) |
| Intercept | 91.60*** | 86.09*** | 85.31*** | 85.08*** |
|  | (88.481,94.713) | (81.336,90.841) | (79.952,90.666) | (79.700,90.453) |
| $R^{2}$ | 0.213 | 0.214 | 0.220 | 0.221 |
| $\hat{\sigma}_{u}$ | 13.668 | 13.650 | 13.573 | 13.563 |
| $\hat{\sigma}_{e}$ | 13.545 | 13.545 | 13.545 | 13.545 |
| $\hat{\rho}$ | 0.50 | 0.50 | 0.50 | 0.50 |
| *Notes*: M1: SII with linear and quadratic trend variable and an interaction between SII and the linear and quadratic trend variable. M2: M1+the big five inventory. M3: M2+ sex, age, region, nationality, children under 16 in household, family status, and household size. M4: M3+income class.  $\hat{\sigma}_{u}$: between-unit standard deviation. $\hat{\sigma}_{e}$: within-unit standard deviation. $\hat{\rho}$: proportion of variance explained by between-unit differences. | | | | |

| **Table 14** Random effects model for absolute educational inequalities (SII) in trust (Number of observations: 15,902; number of individuals: 4,690) | | | | |
| --- | --- | --- | --- | --- |
|  | M1 | M2 | M3 | M4 |
|  | beta | beta | beta | beta |
|  | (95%-CI) | (95%-CI) | (95%-CI) | (95%-CI) |
| SII | -1.718 | -1.806 | 0.962 | -0.851 |
|  | (-5.543,2.107) | (-5.636,2.024) | (-2.904,4.827) | (-4.777,3.075) |
| Trend | -4.028*** | -4.059*** | -4.118*** | -4.099*** |
| (linear) | (-5.343,-2.713) | (-5.374,-2.744) | (-5.432,-2.803) | (-5.413,-2.784) |
| Trend (quadratic) | 0.423* | 0.431* | 0.446* | 0.441* |
|  | (0.035,0.811) | (0.043,0.819) | (0.057,0.834) | (0.053,0.830) |
| SII*Trend (linear) | 1.320 | 1.353 | 1.394 | 1.377 |
|  | (-0.764,3.404) | (-0.731,3.436) | (-0.690,3.477) | (-0.706,3.461) |
| SII*Trend (quadratic) | -0.005 | -0.015 | -0.025 | -0.021 |
|  | (-0.630,0.620) | (-0.639,0.610) | (-0.649,0.600) | (-0.646,0.604) |
| Big Five Inventory |  |  |  |  |
| Extraversion |  | 0.271 | 0.359 | 0.285 |
|  |  | (-0.317,0.860) | (-0.227,0.944) | (-0.300,0.869) |
| Agreeableness |  | 0.043 | 0.008 | 0.128 |
|  |  | (-0.572,0.658) | (-0.618,0.633) | (-0.498,0.754) |
| Conscientiousness |  | 1.020*** | 0.744* | 0.767** |
|  |  | (0.450,1.589) | (0.176,1.312) | (0.201,1.334) |
| Neuroticism |  | 1.074** | 0.405 | 0.377 |
|  |  | (0.346,1.802) | (-0.335,1.145) | (-0.361,1.116) |
| Open-Mindedness |  | 4.195*** | 4.031*** | 4.114*** |
|  |  | (3.491,4.900) | (3.331,4.732) | (3.415,4.814) |
| Gender (ref.: male) |  |  |  |  |
| Female |  |  | 1.674** | 1.910*** |
|  |  |  | (0.660,2.688) | (0.894,2.925) |
| Age |  |  | 0.158*** | 0.164*** |
|  |  |  | (0.111,0.206) | (0.117,0.212) |
| Region (ref.: wester Germany) | |  |  |  |
| Eastern Germany |  |  | -1.831** | -1.402* |
|  |  |  | (-2.937,-0.726) | (-2.517,-0.287) |
| Nationality (ref.: German) |  |  |  |  |
| Others |  |  | -3.519* | -3.136* |
|  |  |  | (-6.630,-0.408) | (-6.242,-0.029) |
| Children under 16 living in household (ref.: no) | | |  |  |
| Yes |  |  | -0.617 | -0.575 |
|  |  |  | (-2.250,1.016) | (-2.204,1.055) |
| Family status (ref.: single) | |  |  |  |
| Married |  |  | 1.374 | 0.590 |
|  |  |  | (-0.198,2.946) | (-1.011,2.190) |
| Divorced/widowed |  |  | -1.724 | -1.891* |
|  |  |  | (-3.564,0.116) | (-3.728,-0.053) |
| Household size (ref.: 1 person) | |  |  |  |
| 2 persons |  |  | -1.121 | -1.656 |
|  |  |  | (-2.822,0.581) | (-3.376,0.064) |
| 3 persons |  |  | -1.458 | -2.352* |
|  |  |  | (-3.545,0.629) | (-4.468,-0.236) |
| 4 persons |  |  | -2.296 | -3.242** |
|  |  |  | (-4.652,0.061) | (-5.624,-0.860) |
| Income (ref.: low) |  |  |  |  |
| Moderate |  |  |  | 1.237 |
|  |  |  |  | (-0.033,2.507) |
| High |  |  |  | 3.677*** |
|  |  |  |  | (2.220,5.133) |
| Intercept | 76.04*** | 57.28*** | 50.87*** | 50.46*** |
|  | (73.617,78.462) | (52.689,61.867) | (45.598,56.149) | (45.177,55.750) |
| $R^{2}$ | 0.014 | 0.044 | 0.065 | 0.069 |
| $\hat{\sigma}_{u}$ | 16.339 | 16.004 | 15.757 | 15.711 |
| $\hat{\sigma}_{e}$ | 9.729 | 9.729 | 9.729 | 9.729 |
| $\hat{\rho}$ | 0.74 | 0.73 | 0.72 | 0.72 |
| *Notes*: M1: SII with linear and quadratic trend variable and an interaction between SII and the linear and quadratic trend variable. M2: M1+the big five inventory. M3: M2+ sex, age, region, nationality, children under 16 in household, family status, and household size. M4: M3+income class.  $\hat{\sigma}_{u}$: between-unit standard deviation. $\hat{\sigma}_{e}$: within-unit standard deviation. $\hat{\rho}$: proportion of variance explained by between-unit differences. | | | | |

| **Table 15** Random effects model for relative educational inequalities (RII) in preventive behaviours (Number of observations: 15,902; number of individuals: 4,690) | | | | |
| --- | --- | --- | --- | --- |
|  | M1 | M2 | M3 | M4 |
|  | beta | beta | beta | beta |
|  | (95%-CI) | (95%-CI) | (95%-CI) | (95%-CI) |
| RII | 20.09*** | 20.46*** | 21.20*** | 19.03*** |
|  | (12.354,27.826) | (12.708,28.213) | (13.425,28.965) | (11.193,26.866) |
| Trend (linear) | 5.350*** | 5.222*** | 5.316*** | 5.384*** |
|  | (2.504,8.197) | (2.375,8.068) | (2.472,8.160) | (2.539,8.228) |
| Trend (quadratic) | -1.426*** | -1.393** | -1.412** | -1.428*** |
|  | (-2.268,-0.584) | (-2.235,-0.550) | (-2.254,-0.571) | (-2.269,-0.586) |
| RII*Trend (linear) | -6.706** | -6.584** | -6.655** | -6.728** |
|  | (-11.222,-2.190) | (-11.100,-2.069) | (-11.166,-2.144) | (-11.240,-2.217) |
| RII*Trend (quadratic) | 1.842** | 1.808** | 1.820** | 1.837** |
|  | (0.486,3.198) | (0.451,3.164) | (0.465,3.175) | (0.481,3.192) |
| Big Five Inventory |  |  |  |  |
| Extraversion |  | 0.476 | 0.120 | 0.037 |
|  |  | (-0.422,1.374) | (-0.764,1.003) | (-0.846,0.920) |
| Agreeableness |  | 1.081* | 0.564 | 0.732 |
|  |  | (0.142,2.020) | (-0.379,1.508) | (-0.213,1.678) |
| Conscientiousness |  | 1.606*** | 1.546*** | 1.579*** |
|  |  | (0.736,2.477) | (0.688,2.404) | (0.722,2.435) |
| Neuroticism |  | 3.594*** | 3.350*** | 3.321*** |
|  |  | (2.481,4.706) | (2.232,4.468) | (2.205,4.438) |
| Open-Mindedness |  | 1.329* | 0.903 | 1.020 |
|  |  | (0.252,2.405) | (-0.155,1.961) | (-0.037,2.078) |
| Gender (ref.: male) |  |  |  |  |
| Female |  |  | 4.143*** | 4.426*** |
|  |  |  | (2.612,5.674) | (2.891,5.960) |
| Age |  |  | 0.045 | 0.052 |
|  |  |  | (-0.027,0.117) | (-0.019,0.124) |
| Region (ref.: wester Germany) | |  |  |  |
| Eastern Germany |  |  | -11.15*** | -10.63*** |
|  |  |  | (-12.824,-9.483) | (-12.313,-8.940) |
| Nationality (ref.: German) |  |  |  |  |
| Others |  |  | 2.145 | 2.594 |
|  |  |  | (-2.562,6.851) | (-2.110,7.298) |
| Children under 16 living in household (ref.: no) | | |  |  |
| Yes |  |  | 0.553 | 0.553 |
|  |  |  | (-1.918,3.025) | (-1.915,3.021) |
| Family status (ref.: single) | |  |  |  |
| Married |  |  | 3.943** | 2.923* |
|  |  |  | (1.566,6.321) | (0.501,5.345) |
| Divorced/widowed |  |  | -0.432 | -0.561 |
|  |  |  | (-3.212,2.347) | (-3.339,2.217) |
| Household size (ref.: 1 person) | |  |  |  |
| 2 persons |  |  | 2.516 | 1.697 |
|  |  |  | (-0.056,5.089) | (-0.906,4.301) |
| 3 persons |  |  | 1.393 | 0.186 |
|  |  |  | (-1.764,4.550) | (-3.019,3.390) |
| 4 persons |  |  | 2.768 | 1.523 |
|  |  |  | (-0.795,6.332) | (-2.083,5.129) |
| Income (ref.: low) |  |  |  |  |
| Moderate |  |  |  | 2.639** |
|  |  |  |  | (0.717,4.561) |
| High |  |  |  | 4.665*** |
|  |  |  |  | (2.461,6.869) |
| Intercept | 86.12*** | 61.70*** | 59.11*** | 58.10*** |
|  | (81.192,91.043) | (53.991,69.417) | (50.516,67.706) | (49.480,66.720) |
| $R^{2}$ | 0.005 | 0.016 | 0.052 | 0.054 |
| $\hat{\sigma}_{u}$ | 23.192 | 22.944 | 22.223 | 22.176 |
| $\hat{\sigma}_{e}$ | 21.241 | 21.241 | 21.241 | 21.241 |
| $\hat{\rho}$ | 0.54 | 0.54 | 0.52 | 0.52 |
| *Notes*: M1: RII with linear and quadratic trend variable and an interaction between RII and the linear and quadratic trend variable. M2: M1+the big five inventory. M3: M2+ sex, age, region, nationality, children under 16 in household, family status, and household size. M4: M3+income class.  $\hat{\sigma}_{u}$: between-unit standard deviation. $\hat{\sigma}_{e}$: within-unit standard deviation. $\hat{\rho}$: proportion of variance explained by between-unit differences. | | | | |

| **Table 16** Random effects model for relative educational inequalities (RII) in risk perception (Number of observations: 15,902; number of individuals: 4,690) | | | | |
| --- | --- | --- | --- | --- |
|  | M1 | M2 | M3 | M4 |
|  | beta | beta | beta | beta |
|  | (95%-CI) | (95%-CI) | (95%-CI) | (95%-CI) |
| RII | 16.53** | 14.51** | 12.41* | 10.79* |
|  | (6.146,26.908) | (4.108,24.920) | (1.942,22.877) | (0.223,21.350) |
| Trend (linear) | 1.706 | 1.726 | 1.904 | 2.002 |
|  | (-2.110,5.523) | (-2.089,5.542) | (-1.912,5.719) | (-1.814,5.819) |
| Trend (quadratic) | -0.322 | -0.327 | -0.367 | -0.39 |
|  | (-1.451,0.807) | (-1.456,0.802) | (-1.496,0.762) | (-1.519,0.739) |
| RII*Trend (linear) | 0.366 | 0.388 | 0.259 | 0.127 |
|  | (-5.688,6.420) | (-5.665,6.441) | (-5.793,6.311) | (-5.926,6.179) |
| RII*Trend (quadratic) | -0.251 | -0.256 | -0.23 | -0.199 |
|  | (-2.069,1.567) | (-2.074,1.562) | (-2.048,1.588) | (-2.016,1.619) |
| Big Five Inventory |  |  |  |  |
| Extraversion |  | 0.357 | -0.0899 | -0.133 |
|  |  | (-0.858,1.572) | (-1.307,1.127) | (-1.351,1.084) |
| Agreeableness |  | 3.044*** | 2.511*** | 2.685*** |
|  |  | (1.774,4.313) | (1.211,3.811) | (1.382,3.988) |
| Conscientiousness |  | 1.972** | 2.142*** | 2.174*** |
|  |  | (0.795,3.149) | (0.960,3.323) | (0.993,3.354) |
| Neuroticism |  | -2.719*** | -2.543** | -2.553** |
|  |  | (-4.223,-1.214) | (-4.084,-1.002) | (-4.093,-1.014) |
| Open-Mindedness |  | -2.050** | -2.271** | -2.150** |
|  |  | (-3.506,-0.595) | (-3.728,-0.813) | (-3.608,-0.691) |
| Gender (ref.: male) |  |  |  |  |
| Female |  |  | 2.791** | 2.996** |
|  |  |  | (0.681,4.901) | (0.881,5.112) |
| Age |  |  | -0.141** | -0.135** |
|  |  |  | (-0.240,-0.042) | (-0.234,-0.036) |
| Region (ref.: wester Germany) | |  |  |  |
| Eastern Germany |  |  | -5.570*** | -5.163*** |
|  |  |  | (-7.871,-3.268) | (-7.488,-2.838) |
| Nationality (ref.: German) |  |  |  |  |
| Others |  |  | -4.093 | -3.767 |
|  |  |  | (-10.577,2.390) | (-10.250,2.716) |
| Children under 16 living in household (ref.: no) | | |  |  |
| Yes |  |  | -1.359 | -1.465 |
|  |  |  | (-4.763,2.046) | (-4.867,1.937) |
| Family status (ref.: single) | |  |  |  |
| Married |  |  | 3.794* | 2.875 |
|  |  |  | (0.518,7.069) | (-0.463,6.214) |
| Divorced/widowed |  |  | 1.197 | 1.266 |
|  |  |  | (-2.633,5.026) | (-2.563,5.096) |
| Household size (ref.: 1 person) | |  |  |  |
| 2 persons |  |  | 2.364 | 1.372 |
|  |  |  | (-1.180,5.908) | (-2.216,4.961) |
| 3 persons |  |  | 1.709 | 0.533 |
|  |  |  | (-2.640,6.058) | (-3.884,4.949) |
| 4 persons |  |  | 6.464** | 5.323* |
|  |  |  | (1.555,11.373) | (0.353,10.294) |
| Income (ref.: low) |  |  |  |  |
| Moderate |  |  |  | 4.565*** |
|  |  |  |  | (1.915,7.214) |
| High |  |  |  | 3.937* |
|  |  |  |  | (0.899,6.974) |
| Intercept | 87.56*** | 89.79*** | 95.67*** | 93.84*** |
|  | (80.950,94.169) | (79.394,100.192) | (83.910,107.426) | (82.041,105.636) |
| $R^{2}$ | 0.009 | 0.018 | 0.028 | 0.029 |
| $\hat{\sigma}_{u}$ | 31.292 | 31.075 | 30.811 | 30.765 |
| $\hat{\sigma}_{e}$ | 28.441 | 28.441 | 28.441 | 28.441 |
| $\hat{\rho}$ | 0.55 | 0.54 | 0.54 | 0.54 |
| *Notes*: M1: RII with linear and quadratic trend variable and an interaction between RII and the linear and quadratic trend variable. M2: M1+the big five inventory. M3: M2+ sex, age, region, nationality, children under 16 in household, family status, and household size. M4: M3+income class.  $\hat{\sigma}_{u}$: between-unit standard deviation. $\hat{\sigma}_{e}$: within-unit standard deviation. $\hat{\rho}$: proportion of variance explained by between-unit differences. | | | | |

| **Table 17** Random effects model for relative educational inequalities (RII) in perceived effectiveness (Number of observations: 15,902; number of individuals: 4,690) | | | | |
| --- | --- | --- | --- | --- |
|  | M1 | M2 | M3 | M4 |
|  | beta | beta | beta | beta |
|  | (95%-CI) | (95%-CI) | (95%-CI) | (95%-CI) |
| RII | -2.953 | -3.226 | -2.339 | -3.793 |
|  | (-11.274,5.368) | (-11.571,5.120) | (-10.733,6.054) | (-12.257,4.671) |
| Trend (linear) | -0.778 | -0.812 | -0.797 | -0.764 |
|  | (-3.861,2.305) | (-3.895,2.271) | (-3.880,2.287) | (-3.848,2.319) |
| Trend (quadratic) | -0.197 | -0.188 | -0.191 | -0.198 |
|  | (-1.109,0.716) | (-1.100,0.725) | (-1.103,0.722) | (-1.111,0.714) |
| RII*Trend (linear) | 1.414 | 1.45 | 1.448 | 1.421 |
|  | (-3.477,6.306) | (-3.441,6.342) | (-3.444,6.339) | (-3.471,6.313) |
| RII*Trend (quadratic) | 0.303 | 0.293 | 0.292 | 0.298 |
|  | (-1.167,1.772) | (-1.176,1.763) | (-1.177,1.762) | (-1.172,1.767) |
| Big Five Inventory |  |  |  |  |
| Extraversion |  | -0.604 | -0.530 | -0.591 |
|  |  | (-1.536,0.327) | (-1.465,0.405) | (-1.527,0.345) |
| Agreeableness |  | 0.750 | 0.933 | 1.033* |
|  |  | (-0.224,1.723) | (-0.065,1.932) | (0.031,2.034) |
| Conscientiousness |  | 1.049* | 1.066* | 1.086* |
|  |  | (0.147,1.951) | (0.159,1.974) | (0.179,1.994) |
| Neuroticism |  | 0.938 | 1.223* | 1.201* |
|  |  | (-0.215,2.092) | (0.039,2.407) | (0.018,2.384) |
| Open-Mindedness |  | 0.493 | 0.460 | 0.529 |
|  |  | (-0.623,1.609) | (-0.660,1.579) | (-0.592,1.650) |
| Gender (ref.: male) |  |  |  |  |
| Female |  |  | -0.698 | -0.507 |
|  |  |  | (-2.319,0.923) | (-2.133,1.119) |
| Age |  |  | 0.042 | 0.046 |
|  |  |  | (-0.034,0.117) | (-0.030,0.122) |
| Region (ref.: wester Germany) | |  |  |  |
| Eastern Germany |  |  | -5.709*** | -5.358*** |
|  |  |  | (-7.477,-3.941) | (-7.145,-3.570) |
| Nationality (ref.: German) |  |  |  |  |
| Others |  |  | 4.125 | 4.429 |
|  |  |  | (-0.857,9.107) | (-0.556,9.414) |
| Children under 16 living in household (ref.: no) | | |  |  |
| Yes |  |  | -1.535 | -1.505 |
|  |  |  | (-4.151,1.081) | (-4.121,1.110) |
| Family status (ref.: single) | |  |  |  |
| Married |  |  | 0.353 | -0.288 |
|  |  |  | (-2.163,2.870) | (-2.855,2.278) |
| Divorced/widowed |  |  | -1.563 | -1.695 |
|  |  |  | (-4.505,1.379) | (-4.639,1.249) |
| Household size (ref.: 1 person) | |  |  |  |
| 2 persons |  |  | -0.517 | -0.961 |
|  |  |  | (-3.240,2.206) | (-3.720,1.798) |
| 3 persons |  |  | -1.228 | -1.963 |
|  |  |  | (-4.570,2.114) | (-5.359,1.433) |
| 4 persons |  |  | -0.344 | -1.122 |
|  |  |  | (-4.116,3.428) | (-4.944,2.699) |
| Income (ref.: low) |  |  |  |  |
| Moderate |  |  |  | 1.051 |
|  |  |  |  | (-0.986,3.088) |
| High |  |  |  | 3.007* |
|  |  |  |  | (0.672,5.343) |
| Intercept | 101.6*** | 94.12*** | 92.35*** | 91.97*** |
|  | (96.314,106.915) | (86.001,102.242) | (83.192,101.509) | (82.776,101.161) |
| $R^{2}$ | 0.001 | 0.002 | 0.010 | 0.011 |
| $\hat{\sigma}_{u}$ | 23.487 | 23.466 | 23.326 | 23.311 |
| $\hat{\sigma}_{e}$ | 23.018 | 23.018 | 23.018 | 23.018 |
| $\hat{\rho}$ | 0.51 | 0.51 | 0.51 | 0.51 |
| *Notes*: M1: RII with linear and quadratic trend variable and an interaction between RII and the linear and quadratic trend variable. M2: M1+the big five inventory. M3: M2+ sex, age, region, nationality, children under 16 in household, family status, and household size. M4: M3+income class.  $\hat{\sigma}_{u}$: between-unit standard deviation. $\hat{\sigma}_{e}$: within-unit standard deviation. $\hat{\rho}$: proportion of variance explained by between-unit differences. | | | | |

| **Table 18** Random effects model for relative educational inequalities (RII) in trust (Number of observations: 15,902; number of individuals: 4,690) | | | | |
| --- | --- | --- | --- | --- |
|  | M1 | M2 | M3 | M4 |
|  | beta | beta | beta | beta |
|  | (95%-CI) | (95%-CI) | (95%-CI) | (95%-CI) |
| RII | -4.287 | -4.401 | -0.288 | -2.934 |
|  | (-9.890,1.316) | (-10.011,1.209) | (-5.950,5.374) | (-8.686,2.818) |
| Trend (linear) | -1.592 | -1.637 | -1.722 | -1.695 |
|  | (-3.515,0.331) | (-3.559,0.285) | (-3.645,0.200) | (-3.617,0.227) |
| Trend (quadratic) | 0.104 | 0.116 | 0.137 | 0.131 |
|  | (-0.464,0.672) | (-0.452,0.683) | (-0.431,0.705) | (-0.437,0.698) |
| RII*Trend (linear) | 2.853 | 2.900 | 2.959 | 2.936 |
|  | (-0.195,5.900) | (-0.147,5.946) | (-0.087,6.006) | (-0.110,5.982) |
| RII*Trend (quadratic) | -0.201 | -0.214 | -0.229 | -0.224 |
|  | (-1.115,0.713) | (-1.128,0.699) | (-1.143,0.685) | (-1.138,0.690) |
| Big Five Inventory |  |  |  |  |
| Extraversion |  | 0.394 | 0.525 | 0.417 |
|  |  | (-0.472,1.260) | (-0.337,1.386) | (-0.444,1.277) |
| Agreeableness |  | 0.074 | 0.024 | 0.200 |
|  |  | (-0.832,0.980) | (-0.896,0.944) | (-0.721,1.122) |
| Conscientiousness |  | 1.490*** | 1.080* | 1.114** |
|  |  | (0.651,2.328) | (0.244,1.916) | (0.280,1.948) |
| Neuroticism |  | 1.603** | 0.615 | 0.575 |
|  |  | (0.531,2.676) | (-0.474,1.705) | (-0.512,1.662) |
| Open-Mindedness |  | 6.158*** | 5.916*** | 6.037*** |
|  |  | (5.122,7.195) | (4.885,6.946) | (5.007,7.066) |
| Gender (ref.: male) |  |  |  |  |
| Female |  |  | 2.464** | 2.808*** |
|  |  |  | (0.971,3.957) | (1.313,4.303) |
| Age |  |  | 0.235*** | 0.244*** |
|  |  |  | (0.165,0.305) | (0.174,0.314) |
| Region (ref.: wester Germany) | |  |  |  |
| Eastern Germany |  |  | -2.728** | -2.101* |
|  |  |  | (-4.356,-1.101) | (-3.743,-0.459) |
| Nationality (ref.: German) |  |  |  |  |
| Others |  |  | -5.191* | -4.631* |
|  |  |  | (-9.771,-0.611) | (-9.205,-0.058) |
| Children under 16 living in household (ref.: no) | | |  |  |
| Yes |  |  | -0.904 | -0.843 |
|  |  |  | (-3.309,1.500) | (-3.242,1.556) |
| Family status (ref.: single) | |  |  |  |
| Married |  |  | 1.982 | 0.838 |
|  |  |  | (-0.333,4.297) | (-1.518,3.194) |
| Divorced/widowed |  |  | -2.540 | -2.784* |
|  |  |  | (-5.248,0.169) | (-5.488,-0.079) |
| Household size (ref.: 1 person) | |  |  |  |
| 2 persons |  |  | -1.619 | -2.399 |
|  |  |  | (-4.124,0.886) | (-4.931,0.133) |
| 3 persons |  |  | -2.153 | -3.457* |
|  |  |  | (-5.226,0.920) | (-6.573,-0.342) |
| 4 persons |  |  | -3.374 | -4.755** |
|  |  |  | (-6.844,0.095) | (-8.263,-1.248) |
| Income (ref.: low) |  |  |  |  |
| Moderate |  |  |  | 1.796 |
|  |  |  |  | (-0.073,3.666) |
| High |  |  |  | 5.366*** |
|  |  |  |  | (3.221,7.511) |
| Intercept | 102.2*** | 74.54*** | 64.99*** | 64.40*** |
|  | (98.622,105.718) | (67.789,81.283) | (57.233,72.750) | (56.622,72.172) |
| $R^{2}$ | 0.001 | 0.031 | 0.052 | 0.057 |
| $\hat{\sigma}_{u}$ | 24.074 | 23.584 | 23.217 | 23.150 |
| $\hat{\sigma}_{e}$ | 14.225 | 14.225 | 14.225 | 14.225 |
| $\hat{\rho}$ | 0.74 | 0.73 | 0.73 | 0.73 |
| *Notes*: M1: RII with linear and quadratic trend variable and an interaction between RII and the linear and quadratic trend variable. M2: M1+the big five inventory. M3: M2+ sex, age, region, nationality, children under 16 in household, family status, and household size. M4: M3+income class.  $\hat{\sigma}_{u}$: between-unit standard deviation. $\hat{\sigma}_{e}$: within-unit standard deviation. $\hat{\rho}$: proportion of variance explained by between-unit differences. | | | | |
